# The role of microRNA-3085 in chondrocyte function

**DOI:** 10.1038/s41598-020-78606-6

**Published:** 2020-12-14

**Authors:** Linh Le, Lingzi Niu, Matthew J. Barter, David A. Young, Tamas Dalmay, Ian M. Clark, Tracey E. Swingler

**Affiliations:** 1 Biomedical Research Centre, School of Biological Sciences, Norwich Research Park, University of East Anglia, Norwich, NR4 7TJ Norfolk UK; 2 Institute of Genetic Medicine, Newcastle University, Newcastle-upon-Tyne, UK; 3 Biotechnology Department, Ho Chi Minh City Open University, Ho Chi Minh City, Vietnam

**Keywords:** Cell biology, Molecular biology, Molecular medicine

## Abstract

MicroRNAs have been shown to play a role in cartilage development, homeostasis and breakdown during osteoarthritis. We previously identified miR-3085 in humans as a chondrocyte-selective microRNA, however it could not be detected by Northern blot. The aim of the current study was to prove that miR-3085 is a microRNA and to investigate the function of miR-3085 in signaling pathways relevant to cartilage homeostasis and osteoarthritis. Here, we confirm that miR-3085 is a microRNA and not another class of small RNA using (1) a pre-miR hairpin maturation assay, (2) expression levels in a Dicer null cell line, and (3) Ago2 pulldown. MicroRNA-3085-3p is expressed more highly in micromass than monolayer cultured chondrocytes. Transfection of miR-3085-3p into chondrocytes decreases expression of *COL2A1* and *ACAN*, both of which are validated as direct targets of miR-3085-3p. Interleukin-1 induces the expression of miR-3085-3p, at least in part via NFκB. In a feed-forward mechanism, miR-3085-3p then potentiates NFκB signaling. However, at early time points after transfection, its action appears to be inhibitory. MyD88 has been shown to be a direct target of miR-3085-3p and may be responsible for the early inhibition of NFκB signaling. However, at later time points, MyD88 knockdown remains inhibitory and so other functions of miR-3085-3p are clearly dominant. TGFβ1 also induces the expression of miR-3085-3p, but in this instance, it exerts a feedback inhibition on signaling with *SMAD3* and *SMAD4* shown to be direct targets. This in vitro analysis shows that miR-3085-3p functions in chondrocytes to induce IL-1-signaling, reduce TGFβ1 signaling, and inhibit expression of matrix genes. These data suggest that miR-3085-3p has a role in chondrocyte function and could contribute to the process of osteoarthritis.

## Introduction

Osteoarthritis (OA) is a degenerative disease of the articular joint, involving degradation of the articular cartilage, subchondral bone sclerosis and the formation of osteophytes^1,2^. Several factors (genetic, developmental, biochemical and biomechanical) impact upon the etiology of OA^1^. Cartilage homeostasis is dependent on the expression of appropriate genes by resident chondrocytes and this is aberrant in OA^3^.

One class of small non-coding RNAs known as microRNAs (miRNAs) have a key role in the regulation of gene expression in human cells. MiRNAs are transcribed as primary transcripts (pri-miRNA) and processed to short stem-loop structures (pre-miRNA) in the nucleus. The pre-miRNA is then processed by the ribonuclease, Dicer, forming two complementary short RNA strands. One of these, the guide strand, is integrated into the RNA-induced silencing complex (RISC), the other, the passenger strand, is degraded^4^. After integration into RISC, miRNAs base pair with their complementary mRNA targets, usually in the 3′UTR^5^ to degrade mRNA or repress translation.

The miRNA pathway has a major role in skeletal development. The conditional knockout of Dicer in limb mesenchyme early in embryonic development leads to the formation of a smaller limb^6^. Dicer-null growth plates show diminished chondrocyte proliferation, with enhanced differentiation to postmitotic hypertrophic chondrocytes. Conditional knockout of Dicer in chondrocytes results in defects in skeletal growth and premature death^7^.

Many miRNAs are regulated during cartilage development, with a number of miRNAs regulated by the key cartilage-specifying transcription factor Sox9 (e.g. miR-140 and miR-455), or regulating Sox9 expression (e.g. miR-675 and miR-145 (see^8^).

MicroRNAs are dysregulated in OA and have a functional effect on disease progression in models of disease^8,9^. Studies comparing expression of miRNAs in normal vs osteoarthritic human cartilage show little congruity, likely because of varying sample groups and this makes interpretation difficult. However, understanding the roles of miRNAs in OA is important and may lead to novel therapy^8^.

MicroRNA-140 is the most studied miRNA to date in cartilage and osteoarthritis. Universal knockout of miR-140 leads to mild dwarfism, probably as a result of impaired chondrocyte proliferation^10,11^. Such deletion of miR-140 in mice predisposed them to the development of age-related OA-like changes and increased cartilage destruction in surgically-induced OA, possibly through direct targeting of Adamts5^10,11^. A number of other targets have been identified and validated in vitro which have the potential to be involved in chondrocyte development and/or cartilage homeostasis^12^.

We recently identified a miRNA in humans, previously sequenced (but uncharacterised) in mice and rats, microRNA-3085. We went on to demonstrate that miR-3085 was expressed selectively in cartilage compared to other tissues and its expression rapidly decreased upon chondrocyte isolation and passage in monolayer culture^13^. MicroRNA-3085 is genomically located within an intron of cartilage acidic protein 1 (CRTAC1, previously called CEP-68), the function of which is unknown^14^. CRTAC1 is expressed in both cartilage and bone tissue, but rapidly lost from osteoblasts on culture^14^ and like miR-3085, its expression is also markedly decreased when chondrocytes are digested from cartilage^15^. CRTAC1 is also reported to be increased in expression in arrays of human OA cartilage (e.g.^16^).

The purpose of the current study was to: (1) prove that miR-3085 was a microRNA and not another type of small RNA; (2) investigate the function of miR-3085 in signaling pathways relevant to cartilage homeostasis and osteoarthritis.

## Materials and methods

### Cell culture

SW1353 chondrosarcoma cells were from the American Type Culture Collection, parental and DLD-1 Dicer null cell lines were from Horizon Discovery and originated from a colorectal adenocarcinoma. Cells were cultured in Dulbecco’s modified Eagle’s medium (DMEM, Thermo Fisher Scientific) with GlutaMAX containing 10% (v/v) fetal bovine serum (Sigma Aldrich), 100 IU/ml penicillin and 100 μg/ml streptomycin, 37 °C, 5% (v/v) CO_2_ under normoxia, as described^17^.

Primary human articular chondrocytes (HACs) were isolated from cartilage of osteoarthritis patients as described^18^ and cultured as above. All experimental protocols were approved by NRES Committee East of England (ref: 08/h0304/85 + 5). All tissue donors gave informed written consent. All methods were performed in accordance with relevant laboratory guidelines and institutional regulations for research using human tissues and body fluids.

For micromass culture^19^, primary HACs were grown in monolayer culture in DMEM high glucose, with GlutaMAX (Thermo Fisher Scientific), containing 10% (v/v) fetal bovine serum (Sigma Aldrich), 100 IU/ml penicillin and 100 μg/ml streptomycin (Thermo Fisher Scientific) (growth medium) at 37 °C, 5% (v/v) CO_2_ until passage two. Confluent cells were then trypsinised and resuspended at a density of 2 × 10^7^ in growth medium. Micromass was obtained by pipetting 20 μl cell suspension into individual wells of 24-well plates and incubating for 3 h to attach. One ml growth medium was then added, and the micromass was incubated for 24 h. Growth medium was then replaced with DMEM high glucose, with GlutaMax (Thermo Fisher Scientific) containing 1 × insulin-transferrin-selenium (Thermo Fisher Scientific) and 0.5% (v/v) fetal bovine serum (Sigma Aldrich), with or without recombinant human TGFβ1 (4 ng/ml) (R&D Systems) or IL-1β (5 ng/ml) (First Link (UK) Ltd) for 24 h. Inhibition of NFκB was achieved using JSH-23 at 10 μM (Calbiochem). JSH-23 was added 1 h before adding IL-1β and was kept in culture media for another 24 h.

### Transient transfection

The 3′UTR of mRNAs containing the predicted binding site of miR-3085-3p were subcloned into pmirGLO (Promega), using QuikChange II XL site-directed mutagenesis kit (Agilent) to introduce mutations. Constructs were sequence verified (Source Bioscience). SW1353 cells were seeded into 96-well plate wells at 5 × 10^4^ cells/ml in 100 ul medium overnight and transiently transfected with 100 ng reporter plasmid, 50 nM miR-3085-3p mimic (Qiagen) or non-targeting control (Negative Control miRCURY LNA miRNA Mimic, Qiagen) using Lipofectamine 3000 (Thermo Fisher Scientific), according to manufacturer’s instructions for either 24 h or 48 h (see^20^). Cell lysates were assayed for luciferase using the Dual Luciferase Reporter Assay Kit (Promega), read with an EnVision 2103 Multilabel plate reader (Perkin Elmer). Relative luciferase activity was the ratio of firefly luciferase to Renilla luciferase activity^20^.

Signalling pathways were measured using p(CAGA)_12_-luc (Smad2/3)^21^; κB-luc (NFκB)^22^; TOPFlash (canonical Wnt) reporters^23^. 100 ng of the plasmid and 10 ng of constitutive Renilla plasmid were co-transfected into SW1353 cells with 50 nM miR-3085-3p mimic or non-targeting control. After serum starvation for 24 h, cells were treated with either TGFβ1 (4 ng/ml) (R&D Systems) or IL-1β (5 ng/ml) (First Link (UK) Ltd) for 6 h. Luciferase activity and data analysis were as described above.

HACs were plated in 96-well plate wells at a density of 7 × 10^4^ cells/ml in 100 µl, and grown to 80%-90% confluence. MicroRNA-3085-3p mimic (50 nM), non-targeting control (50 nM), MyD88 siRNA (50 nM) and negative control siRNA (50 nM) (AllStars Negative Control siRNA, Qiagen) were transfected as described above and^20^. Cells were then incubated at 37 °C in 5% CO_2_ for 48 h. After serum starvation for 24 h, cells were treated with recombinant human TGFβ1 (4 ng/ml) (R&D Systems), IL-1β (5 ng/ml) (First Link (UK) Ltd) for another 8 h^20^, followed by RNA isolation.

### RNA isolation and qRT-PCR

Trizol reagent (Thermo Fisher Scientific) was used to isolate total RNA from cultured cells according to manufacturer’s instructions. The miRCURY LNA Universal cDNA synthesis kit (Exiqon) and miRNA-specific LNA primers (Exiqon) was used for quantification of mature miRNA transcripts by qRT-PCR. Data were normalized to U6 as the housekeeping gene. For mRNA, Ambion Cells-to-cDNA II Kit (Life Technologies) or SuperScript II RT (Thermo Fisher Scientific) was used with data normalised to expression of 18S rRNA ribosomal RNA. Only samples where the housekeeping genes are expressed within median + /− 1 Ct are included in analyses. Primer sequences are listed in Supplementary Table 1. Both miRNA and mRNA relative quantifications were calculated using the ∆∆CT method. Fluorescence for each cycle was analysed by the real-time PCR 7500 system (Applied Biosystems)^20^.

### Western blot

SW1353 cells were plated in 6-well plate wells at 1.5 × 10^5^ cells/well and left to adhere overnight. Cells were transiently transfected with 50 nM miR-3085-3p mimic (Qiagen), siRNA (Qiagen) or non-targeting controls (Qiagen) for 48 h. After serum starvation for another 24 h, cells were stimulated with IL-1β (5 ng/ml) (First Link (UK) Ltd) for 30 min or TGFβ1 (4 ng/ml) (R&D Systems) for 2 h and washed twice in ice-cold phosphate buffered saline (PBS). Whole cell lysates were harvested into ice cold RIPA buffer (50 mM Tris–HCL pH7.6, 150 mM NaCl, 1% (v/v) Triton x-100, 1% (w/v) sodium deoxycholate, 0.1% (w/v) SDS, 10 mM NaF, 2 mM Na_3_VO_4_, 1 × protease inhibitor cocktail III (Thermo Fisher Scientific)). Samples were separated on reducing SDS-PAGE, transferred to PVDF membrane and probed overnight at 4 °C. The p65 (#8242), phospho-p65 (#3033), IκBα (#4814), phosphor-IκBα (#2859),SMADs (#3103, #9513, #38,454), phospho-SMADs (#9523), MyD88 (#4283), β-catenin (#9582), phospho-β-catenin (#9561) and GAPDH (#2118) (all from Cell Signaling Technology, used at recommended concentrations) were detected using HRP-conjugated secondary antibodies (DAKO), visualised using Pierce ECL Western Blotting Substrate (Thermo Fisher Scientific), and imaged by ChemiDoc MP Imaging System (Biorad)^13^.

### Cellular fractionation

SW1353 cells were plated in 60-mm culture dishes at 3.5 × 10^5^ cells/dish overnight. The cells were then transiently transfected with either miR-3085-3p mimic (Qiagen) or non-targeting control (Qiagen), serum starved, and treated with IL-1β or TGFβ1 as described above. Nuclear and cytosolic fractions were purified using the Nuclear and Cytoplasmic Extraction kit (Thermo Fisher Scientific) according to manufacturer’s instructions, using 1 × protease inhibitor cocktail III (Thermo Fisher Scientific), 1 × phosphatase inhibitor cocktail 2 (Sigma Aldrich), and 1 × phosphatase inhibitor cocktail 3 (Sigma Aldrich).

### Hairpin maturation assay

An approximately 500 bp region containing the precursor of miR-140 or miR-3085 sequences was sub-cloned into pcDNA3.0. Primers for sub-cloning miR-3085 were.

- 5′-ATGCAAGCTTAGGATCAAGAGCAGGATTGG-3′,
- 5′-ATGCAAGCTTCTGGCCTCAGAGAAGACTGG-3′ and for miR-140 were
- 5′-ATGCAAGCTTAGAGAGAGAGAGCGCTGTGG-3′,
- 5′-ATGCAAGCTTGCAACACTCTTGCACTTTGC-3′.

SW1353 cells were transiently transfected using Lipofectamine 3000 (Thermo Fisher Scientific) according to manufacturer’s instructions) with expression constructs for either precursor miR-140 or precursor miR-3085 compared to an empty vector and cultured for 48 h. RNA was isolated and miRCURY LNA Universal cDNA synthesis kit (Exiqon) and miRNA-specific LNA primers (Exiqon) for U6, miR-3085-3p and miR-140-5p were used for quantification of mature miRNA transcripts by qRT-PCR.

### Agonaute pulldown

SW1353 cells were transiently transfected with either precursor miR-140 or precursor miR-3085 for 48 h as above. Cell lysates were harvested with 0.5%(v/v) NP40, 150 mM KCl, 25 mM Tris–glycine pH7.5, 2 mM EDTA, 0.5 mM DTT, and 1 × protease inhibitor cocktail III (Thermo Fisher Scientific). Samples were pulled down with Ago2 antibody (MABE253, Sigma Aldrich) using Dynabeads Protein G (Thermo Fisher Scientific) and DynaMag Magnet at 4 °C, overnight. In order to quantify miRNA expression, targeted Ago2 was eluted with Cells-to-cDNA II lysis buffer (Thermo Fisher Scientific) and the miRCURY LNA Universal cDNA synthesis kit (Exiqon, Denmark) and miRNA-specific LNA primers (Exiqon, Denmark) for miR-140-3p, miR-29b-3p, and miR-3085-3p were used for quantification of mature miRNA transcripts by qRT-PCR.

### Statistical analysis

Data were tested for normal distribution and analysed using Student’s *t*-test to compare between two samples, or one-way ANOVA with post-hoc Tukey’s test to compare between multiple samples using GraphPad Prism version 6. Experiments were performed on three independent replicates (n = 3); for primary human chondrocytes, three different patient isolates were used.

## Results

### MicroRNA-3085 is a microRNA

**Figure 1:**
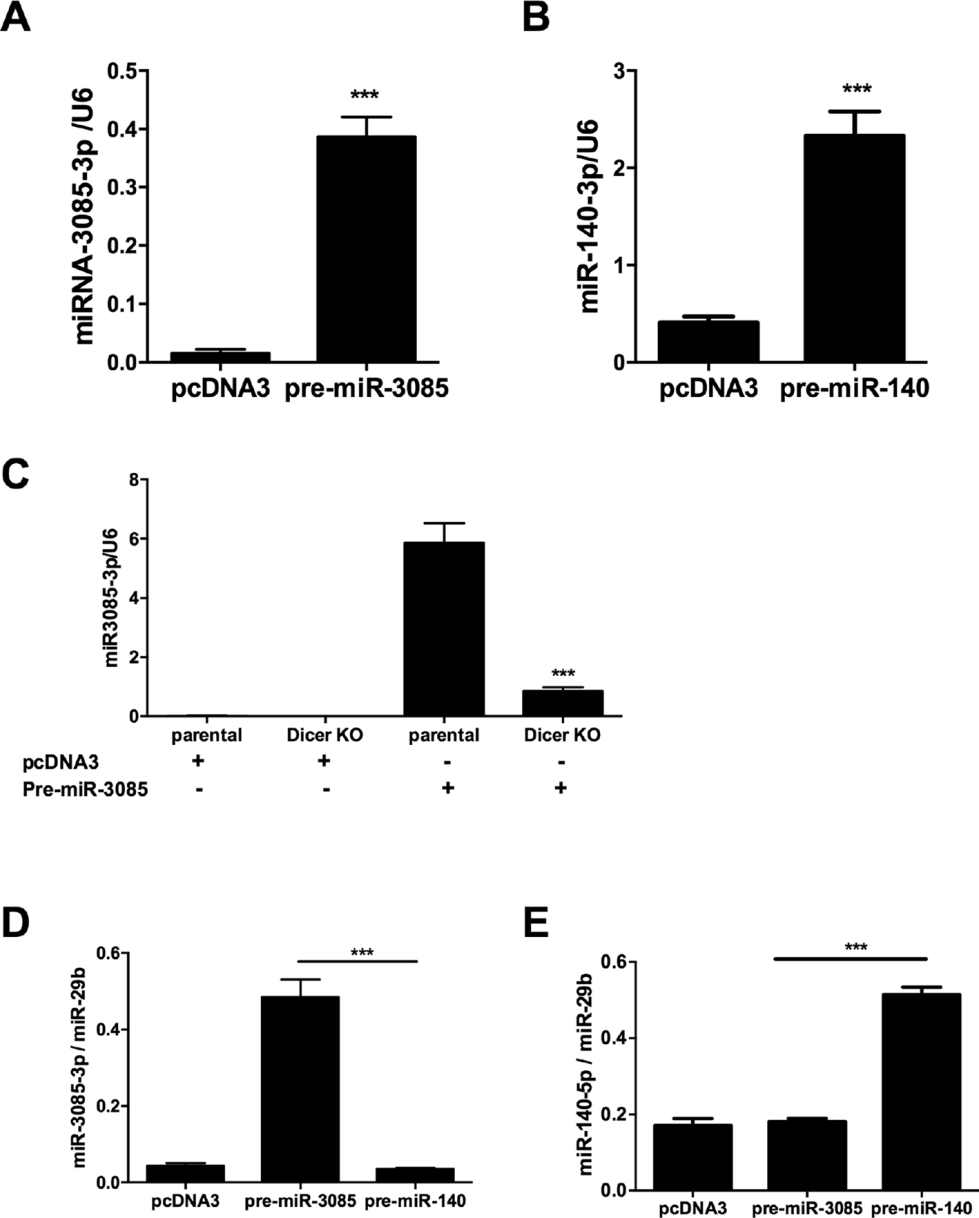
miR-3085 is a microRNA. (**A**) SW1353 chondrosarcoma cells were transiently transfected with expression plasmids for either (**A**). pre-miR-3085 hairpin or (**B**). pre-miR-140 hairpin and empty vector (pcDNA3) control and cultured for 48 h. Expression of mature miR-3085-3p or miR-140-3p was measured by qRT-PCR. (**C**) Parental DLD-1 cells or isogenic Dicer null (KO) cells were transiently transfected with expression plasmids for pre-miR-3085 hairpin and culture for 48 h. Expression of mature miR-3085-3p was measured by qRT-PCR. (**D**,**E**). SW1353 cells were transiently transfected with either pre-miR-3085 or pre-miR-140 for 48 h, cell lysates were immunoprecipitated using an Ago2 antibody; mature miR-3085-3p, miR-140-3p and miR-29b-3p were measured by qRT-PCR. Mean + /− SEM, n = 3; (**A**,**B**) Student’s t-test; (**C**–**E**), ANOVA with Tukey’s post test; **p < 0.01; ***p < 0.001.

Northern blot of chondrocyte RNA probed for miR-3085 failed to show a signal^13^. In order to verify the identity of this RNA as a microRNA, an expression construct of the presumed pre-miR-3085 hairpin sequence was transiently transfected into SW1353 cells. This lead to a statistically significant increase in the mature miR-3085-3p (*p* < 0.001, Fig. 1A). As a positive control, the same increase in mature miR-140-3p was measured after transfection with pre-miR-140 expression construct (Fig. 1B). Furthermore, this increase in mature miR-3085-3p was significantly reduced in DLD-1 Dicer null cells compared to isogenic wild-type cells (*p* < 0.001, Fig. 1C). Finally, mature miR-3085-3p was immunoprecipitated with an anti-AGO2 antibody after transfection with pre-miR-3085 (but not pre-miR-140) and vice versa (Fig. 1D,E). Western blot of the immunoprecipitates with anti-AGO2 shows equal loading (Supplementary Fig. 1).

### MicroRNA-3085-3p modulates the expression of matrix genes

**Figure 2:**
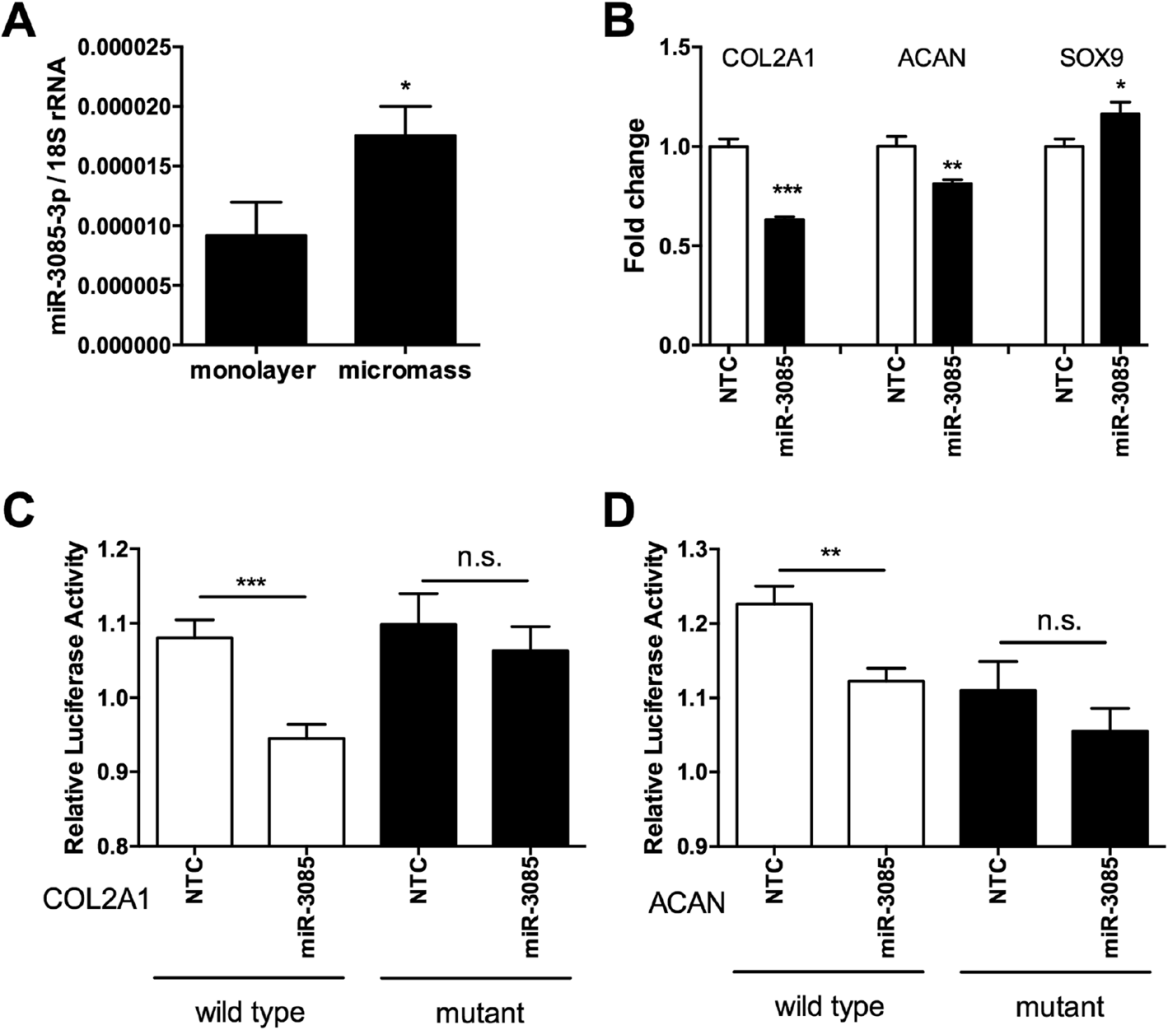
miR-3085 downregulates type II collagen and aggrecan expression. (**A**) Primary human articular chondrocytes were cultured in micromass culture for 48 h; miR-3085-3p was measured by qRT-PCR. (**B**) Micromass cultures of primary human articular chondrocytes transfected with miR-3085-3p mimic or non-targeting control (NTC) for 48 h; COL2A1 and ACAN were measured by qRT-PCR. SW1353 cells were transiently transfected with the (**C**). ACAN 3′UTR and (**D**). COL2A1 3′UTR subcloned into the pmirGLO vector (wild-type) or a construct with miR-3085-3p seed sites mutated (mutant) with miR-3085-3p mimic or non-targeting control (NTC) for 24 h. Firefly luciferase relative light units were normalised to Renilla relative light units to give overall relative light units. Mean + /− SEM, n = 3; Student’s t-test; *p < 0.05; **p < 0.01; ***p < 0.001.

The expression of miR-3085-3p is increased by micromass (three-dimensional) culture of human articular chondrocytes (*p* < 0.05, Fig. 2A), which significantly enhances expression of matrix genes^19^. However, the overexpression of miR-3085 represses the expression of both *COL2A1* (*p* < 0.001) and *ACAN *(*p* < 0.01), though *SOX9* expression shows a small increase (*p* < 0.05) (Fig. 2B). Expression of luciferase controlled by the 3′UTR of either the *COL2A1* or *ACAN* gene shows that miR-3085-3p significantly reduces this expression and that this is rescued by mutation of the miR-3085-3p seed site in each UTR (Fig. 2C,D). This shows that these genes are direct targets of miR-3085-3p.

### MicroRNA-3085-3p and NFκB signaling

**Figure 3:**
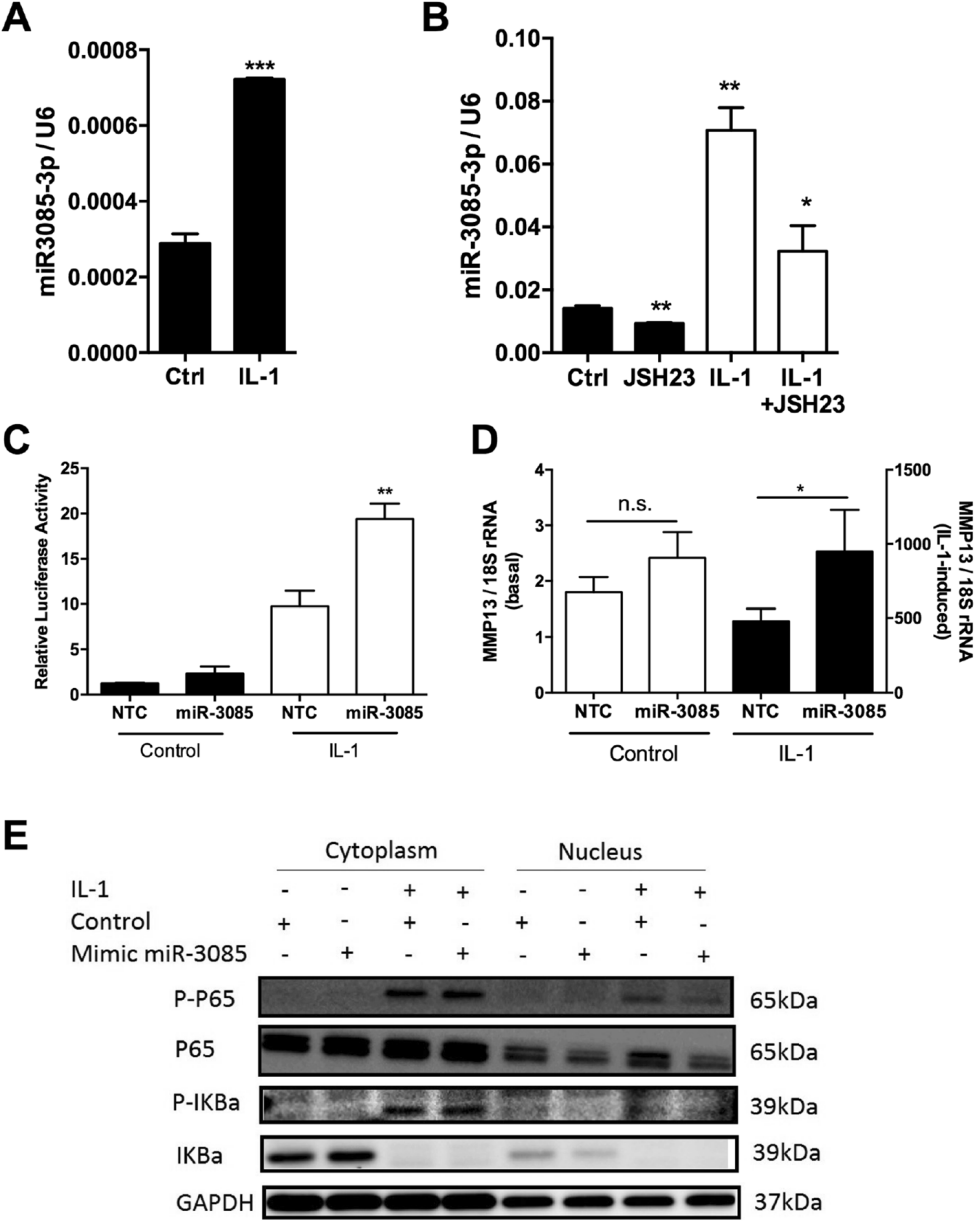
IL-1 induces miR-3085-3p which represses NFκB signaling. Primary human articular chondrocytes were cultured in micromass culture for 48 h. (**A**) cells were then treated with IL-1β (5 ng/ml) or control for 24 h; (**B**) cells were then treated with IL-1β (5 ng/ml) or control + /− an NFκB inhibitor JSH-23 (10 μM) or vehicle 24 h; miR-3085-3p was measured by qRT-PCR. (**C**) SW1353 cells were transfected with an NFκB luciferase reporter + /− miR-3085-3p mimic or non-targeting control (NTC) for 24 h prior to stimulation with IL-1β (5 ng/ml) or control for 8 h. (**D**) Primary human articular chondrocytes were grown in monolayer culture and transiently transfected with miR-3085-3p or a non-targeting control (NTC) for 24 h prior to stimulation with IL-1β (5 ng/ml) or control for 6 h; *MMP13* was measured by qRT-PCR. Mean + /− SEM, n = 3; Student’s t-test; *p < 0.05; **p < 0.01; ***p < 0.001. (**E**) SW1353 cells were grown in monolayer culture and transiently transfected with miR-3085-3p or a non-targeting control for 48 h, serum starved for 24 h and stimulated with IL-1β (5 ng/ml) or control for 30 min, fractionation and western blot analysis. Full-length blots are presented in Supplementary data.

Interleukin-1 and NFκB signaling are deemed key in OA^1,24^. Interleukin-1β induces expression of miR-3085-3p in primary human articular chondrocytes in either monolayer or micromass culture (*p* < 0.001, Fig. 3A, micromass) and this is in part dependent on NFκB signaling (Fig. 3B). IL-1-induced activation of an NFκB reporter, transiently transfected into SW1353 cells is further increased by co-transfection with miR-3085-3p (*p* < 0.01, Fig. 3C), with the same pattern seen for IL-1 induced *MMP13* expression in HACs (*p* < 0.05, Fig. 3D), at 8 or 6 h of induction respectively. However, at an optimal early time point (30 min), miR-3085-3p mimic decreased nuclear levels of IL-1-induced p65 and phospho-p65 (Fig. 3E). Indeed, time course data from qRT-PCR of IL-1 induced *MMP13* expression did not show a response until 6 h of stimulation (Supplementary Fig. 2).

**Figure 4:**
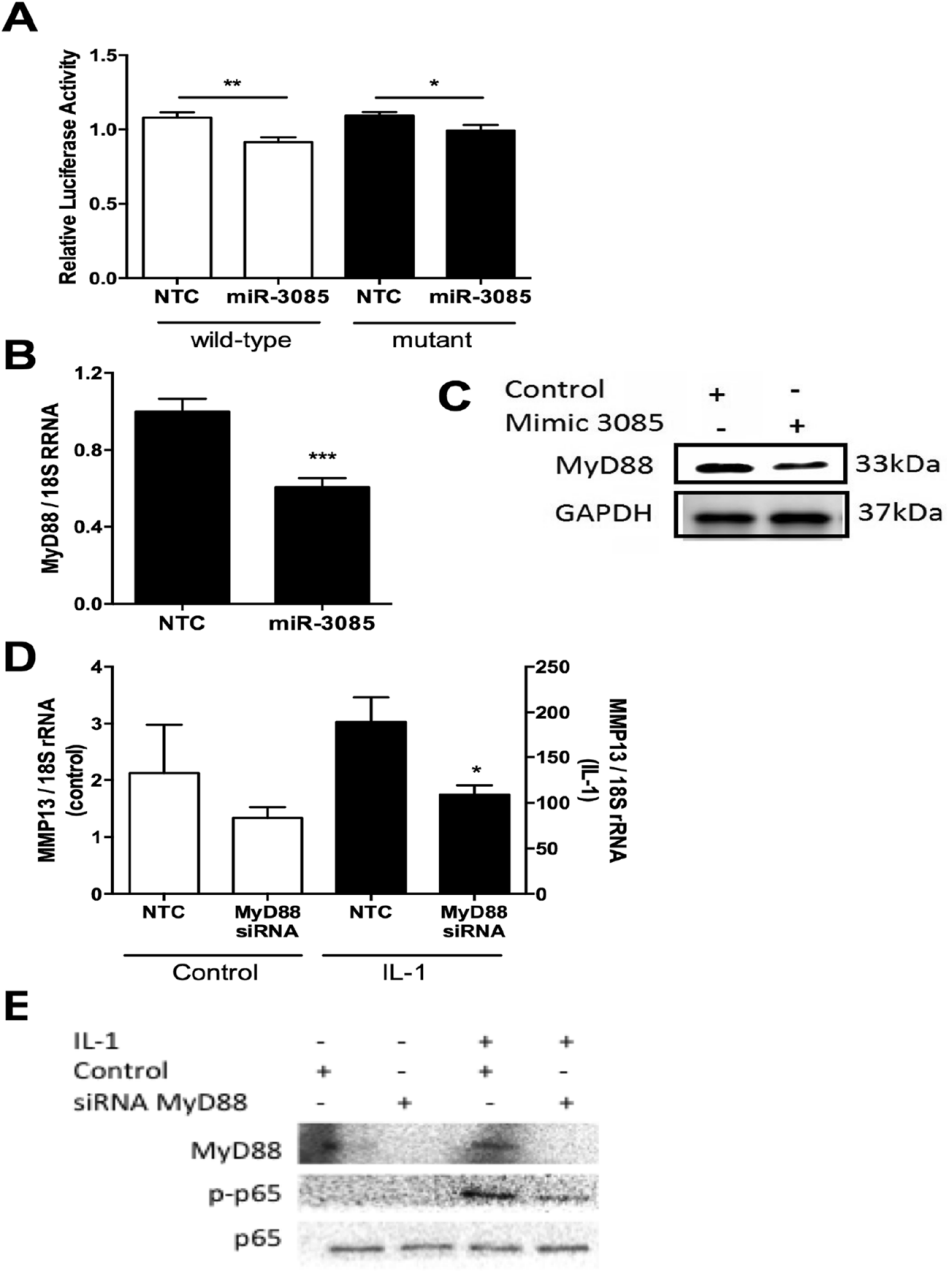
MyD88 is a direct target of miR-3085-3p. (**A**) SW1353 cells were transiently transfected with the MyD88 3′UTR subcloned into the pmirGLO vector (wild-type) or a construct with miR-3085-3p seed sites mutated (mutant) with miR-3085-3p mimic or non-targeting control (NTC) for 24 h. Firefly luciferase relative light units were normalised to Renilla relative light units to give overall relative light units. (**B**) Primary human articular chondrocytes were grown in monolayer culture and transiently transfected with miR-3085-3p or a non-targeting control (NTC) for 48 h; *MyD88* was measured by (**B**) qRT-PCR and (**C**) SW1353 cells were transfected with miR-3085-3p or a non-targeting control (NTC) for 48 h, MyD88 protein level were measured by western blot. (**D**) Primary human articular chondrocytes were grown in monolayer culture and transiently transfected with MyD88 siRNA or a non-targeting control (NTC) for 24 h prior to stimulation with IL-1β (5 ng/ml) or control for 6 h; *MMP13* was measured by qRT-PCR. Mean + /- SEM, n = 3. Student’s t-test; *p < 0.05; **p < 0.01; ***p < 0.001. (**E**) SW1353 cells were grown in monolayer culture and transiently transfected with MyD88 siRNA or a non-targeting control (NTC) for 24 h prior to stimulation with IL-1β (5 ng/ml) or control for 30 min prior to western blot analysis. Full-length blots are presented in Supplementary data; N.B. the full blot for MyD88 in (**C**) is over saturated.

Since MyD88 is an adapter protein involved in IL-1 signalling^25^ and *MyD88* is predicted to be a direct target of miR-3085-3p, we explored this further. MyD88 is experimentally shown to be a direct target of miR-3085-3p, though rescue by mutation of the seed site is partial rather than complete (Fig. 4A). Quantitative RT-PCR (Fig. 4B) and western blot (Fig. 4C) also demonstrates that overexpression of miR-3085-3p leads to a decrease in MyD88 mRNA (*p* < 0.001) and protein. Treatment with siRNA against *MyD88* leads to a decrease in phospho-p65 (Fig. 4D) and it also decreases IL-1-induced *MMP13* expression (*p* < 0.05, Fig. 4E), so this is not the mechanism by which miR-3085-3p potentiates IL-1/ NFκB signaling.

### MicroRNA-3085-3p and Smad signaling

**Figure 5:**
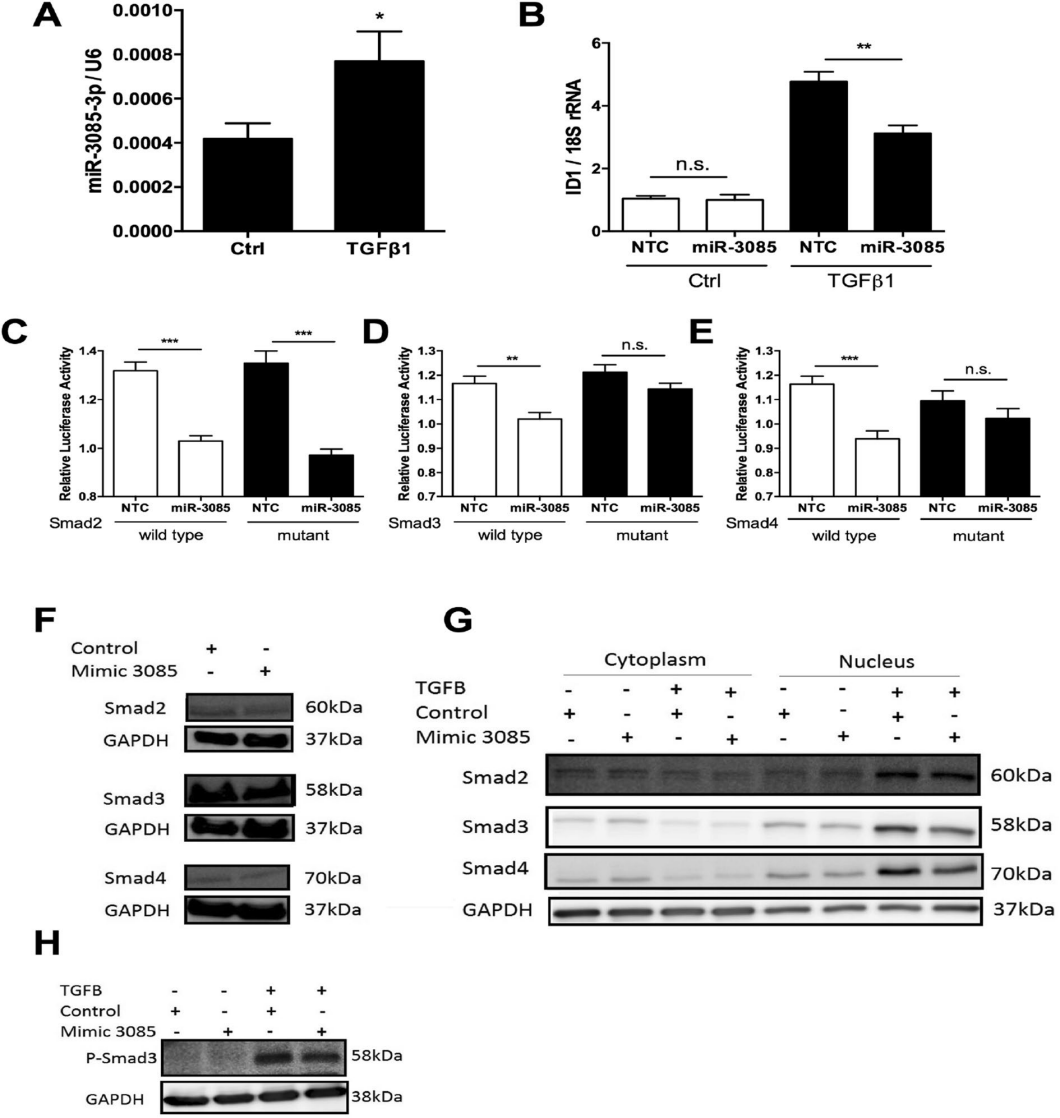
TGFβ induces miR-3085-3p which represses Smad signaling. Primary human articular chondrocytes were cultured in micromass culture for 48 h. (**A**) cells were then treated with TGFβ1 (4 ng/ml), or control for 24 h; miR-3085-3p was measured by qRT-PCR. (**B**) Primary human articular chondrocytes were grown in monolayer culture and transiently transfected with miR-3085-3p or a non-targeting control (NTC) for 24 h prior to stimulation with TGFβ1 (4 ng/ml), or control for 6 h; *ID1* was measured by qRT-PCR. (**C**–**E**) SW1353 cells were transiently transfected with the (**C**) Smad2, (**D**) Smad3, (**E**) Smad4. 3′UTR subcloned into the pmirGLO vector (wild-type) or a construct with miR-3085-3p seed sites mutated (mutant) with miR-3085-3p mimic or non-targeting control (NTC) for 24 h. Firefly luciferase relative light units were normalised to Renilla relative light units to give overall relative light units. Mean + /− SEM, n = 3; Student’s t-test; *p < 0.05; **p < 0.01; ***p < 0.001. (**F**) SW1353 cells were transiently transfected with miR-3085-3p or a non-targeting control for 48 h prior to western blot analysis. (**G**) SW1353 cells were grown in monolayer culture and transiently transfected with miR-3085-3p or a non-targeting control for 48 h prior to stimulation with TGFβ1 (4 ng/ml), or control for 2 h, fractionation and western blot analysis. (**H**) SW1353 cells were grown in monolayer culture and transiently transfected with miR-3085-3p or a non-targeting control for 48 h prior to stimulation with TGFβ1 (4 ng/ml), or control for 2 h and western blot analysis. Full-length blots are presented in Supplementary data.

TGFβ-signalling is also key in OA^1,24^. Similar to IL-1, TGFβ1 also induces expression of miR-3085-3p (*p* < 0.05, Fig. 5A). In this instance, miR-3085-3p does not show a significant effect on TGFβ1-induced luciferase from a Smad-responsive luciferase reporter (CAGA_12_) (Supplementary Fig. 3). TGFβ1-induced expression of the inhibitor of DNA-binding 1 (*ID1)* gene is repressed by over-expression of miR-3085-3p (*p* < 0.01, Fig. 5B). *SMAD3* and *SMAD4* are direct targets for miR-3085-3p; a *SMAD2* 3′ UTR construct is also repressed by miR-3085-3p, but this repression is not rescued by mutation of the seed site (Fig. 5C–E). In total cell extracts, miR-3085-3p reduced expression of SMAD2, SMAD3 and SMAD4 protein (Fig. 5F), whilst in fractionated cells these SMADs were decreased in the nuclear fraction (Fig. 5G) and phospho-SMAD3 was also reduced in whole cell extracts (Fig. 5H).

## Discussion

MicroRNA-3085 was identified in humans during a small RNA-Seq experiment in primary human articular chondrocytes from osteoarthritis patients^13^ where it was shown to be genomically located in the final intron for CRTAC1, a gene expressed in cartilage. It had previously been identified in mouse and rat genomes, but was intergenic in these, though new annotation now shows it also overlaps with CRTAC1 in the rat genome (Ensembl Release 92, data not shown).

In northern blot experiments in RNA purified from cells, expression was undetectable^13^. It has previously been noted that poorly expressed microRNAs require validation via more sensitive methodologies^26^. We therefore looked at its identity as a miRNA using three experiments: transient transfection of an expression plasmid for the hairpin precursor sequence and measuring the mature miR-3085-3p^27,28^; an identical experiment comparing isogenic cell lines which are wild-type or Dicer null^29^; pull down with anti-Argonaute antibody^30^. In each case, miR-3085 behaved in an identical manner to a miR-140 control, providing strong evidence that it is a miRNA rather than any other species of small RNA.

Expression of miR-3085-3p increases when cells are grown in micromass culture compared to monolayer. This is a similar pattern to CRTAC-1 itself^31^, suggesting that they may be regulated by the same promoter. However, overexpression of Sox9, the master regulator of the chondroctye phenotype, significantly induces *CRTAC1* expression but has a lesser effect on miR-3085-3p expression (data not shown). MicroRNA-3085-3p increases expression of *SOX9* in primary HACs in micromass culture, but decreases expression of both *COL2A1* and *ACAN* genes. These genes are direct targets of miR-3085-3p and this direct effect appears to be dominant in regulating *COL2A1* and *ACAN* expression.

Interleukin-1 induces expression of miR-3085-3p and this is in part mediated by NFκB signaling. Interleukin-1 induction of an NFκB -induced luciferase reporter is further induced by overexpression of miR-3085-3p and this is reinforced by a similar effect on the IL-1-induced expression of the *MMP13* gene. Whilst we have identified MyD88 as a direct target of miR-3085-3p, we have excluded its role in the effect of the microRNA on IL-1 signaling. The direct target of miR-3085-3p responsible has not been identified either using prediction software or by experimental overexpression of miR-3085-3p followed by microarray^13^.

Transforming growth factor beta also induces expression of miR-3085-3p, however the miRNA has no significant effect on the (CAGA) _12_ Smad sensitive reporter. This is difficult to explain, since miR-3085-3p clearly decreases expression of Smads 2–4 at both mRNA and protein level. However, the TGFβ1 -induced expression of *ID1*, used as a model TGFβ1-inducible gene, was repressed by the overexpression of miR-3085-3p. Interestingly, two other genes often used to demonstrate induction by TGFβ1, *PAI1* and *TIMP3*, are both predicted to be direct targets of miR-3085-3p and therefore not useful in this context.

The effect of miR-3085-3p on IL-1 signalling represents a feed-forward mechanism, whilst on TGFβ-signalling, it is feed-back. Both of these have been described for microRNAs previously^32^.

Some of the experiments reported have been performed in the SW1353 chondrosarcoma cell line and not in primary HACs. Whilst this is a limitation, the addition of exogenous miR-3085-3p to cell lines does demonstrate the impact of the miRNA on signaling pathways and their outcome. For experiments requiring transient transfection of plasmid DNA, we find that its efficiency in HACs is low and this restricts its utility to cell lines.

These data strongly suggest that miR-3085 functions in cartilage signaling and homeostasis. We have shown that it decreases matrix gene expression, enhances IL-1 signalling and decreases TGFβ signaling, suggesting that it acts to promote catabolism. However, since miRNAs regulate the expression of a number of genes and pathways, this would be best investigated in vivo. Whilst we were able to gain founder mice with deletions of miR-3085 using CrispR-Cas9, we could not breed these forwards to germline. We are not certain if this is simply a technical issue or whether the miRNA has a more critical function in development.

## Supplementary information

Supplementary information.

## References

[1] Goldring SR, Goldring MB (2006). Clinical aspects, pathology and pathophysiology of osteoarthritis. J. Musculoskelet. Neuronal. Interact..

[2] Hunter DJ, Bierma-Zeinstra S (2019). Osteoarthritis. Lancet.

[3] Fisch KM (2018). Identification of transcription factors responsible for dysregulated networks in human osteoarthritis cartilage by global gene expression analysis. Osteoarthr. Cartil..

[4] O'Brien J, Hayder H, Zayed Y, Peng C (2018). Overview of MicroRNA biogenesis, mechanisms of actions, and circulation. Front. Endocrinol. (Lausanne).

[5] Winter J, Jung S, Keller S, Gregory RI, Diederichs S (2009). Many roads to maturity: microRNA biogenesis pathways and their regulation. Nat. Cell Biol..

[6] Harfe BD, McManus MT, Mansfield JH, Hornstein E, Tabin CJ (2005). The RNaseIII enzyme Dicer is required for morphogenesis but not patterning of the vertebrate limb. Proc. Natl. Acad. Sci. USA.

[7] Kobayashi T (2008). Dicer-dependent pathways regulate chondrocyte proliferation and differentiation. Proc. Natl. Acad. Sci. USA.

[8] Swingler TE (2019). The function of microRNAs in cartilage and osteoarthritis. Clin. Exp. Rheumatol..

[9] Malemud CJ (2018). MicroRNAs and osteoarthritis. Cells.

[10] Miyaki S (2010). MicroRNA-140 plays dual roles in both cartilage development and homeostasis. Genes Dev..

[11] Nakamura Y, Inloes JB, Katagiri T, Kobayashi T (2011). Chondrocyte-specific microRNA-140 regulates endochondral bone development and targets Dnpep to modulate bone morphogenetic protein signaling. Mol. Cell. Biol..

[12] Le LT, Swingler TE, Clark IM (2013). Review: the role of microRNAs in osteoarthritis and chondrogenesis. Arthritis Rheum..

[13] Crowe N (2016). Detecting new microRNAs in human osteoarthritic chondrocytes identifies miR-3085 as a human, chondrocyte-selective, microRNA. Osteoarthr. Cartil..

[14] Steck E (2001). Chondrocyte expressed protein-68 (CEP-68), a novel human marker gene for cultured chondrocytes. Biochem. J..

[15] Benz K, Breit S, Lukoschek M, Mau H, Richter W (2002). Molecular analysis of expansion, differentiation, and growth factor treatment of human chondrocytes identifies differentiation markers and growth-related genes. Biochem. Biophys. Res. Commun..

[16] Ijiri K (2008). Differential expression of GADD45beta in normal and osteoarthritic cartilage: potential role in homeostasis of articular chondrocytes. Arthritis Rheum..

[17] Swingler TE (2012). The expression and function of microRNAs in chondrogenesis and osteoarthritis. Arthritis Rheum..

[18] Culley KL (2013). Class I histone deacetylase inhibition modulates metalloproteinase expression and blocks cytokine-induced cartilage degradation. Arthritis Rheum..

[19] Greco KV (2011). High density micromass cultures of a human chondrocyte cell line: a reliable assay system to reveal the modulatory functions of pharmacological agents. Biochem. Pharmacol..

[20] Le LT (2016). The microRNA-29 family in cartilage homeostasis and osteoarthritis. J. Mol. Med. (Berlin).

[21] Pais H (2010). Analyzing mRNA expression identifies Smad3 as a microRNA-140 target regulated only at protein level. RNA.

[22] Davidson RK (2013). Sulforaphane represses matrix-degrading proteases and protects cartilage from destruction in vitro and in vivo. Arthritis Rheum..

[23] Korinek V (1997). Constitutive transcriptional activation by a beta-catenin-Tcf complex in APC-/- colon carcinoma. Science.

[24] Goldring MB (2006). Update on the biology of the chondrocyte and new approaches to treating cartilage diseases. Best Pract. Res. Clin. Rheumatol..

[25] Gabay C, Lamacchia C, Palmer G (2010). IL-1 pathways in inflammation and human diseases. Nat. Rev. Rheumatol..

[26] Grad Y (2003). Computational and experimental identification of *C. elegans* microRNAs. Mol. Cell.

[27] Fan J (2019). A simplified system for the effective expression and delivery of functional mature microRNAs in mammalian cells. Cancer Gene Ther..

[28] Qu B, Han X, Tang Y, Shen N (2012). A novel vector-based method for exclusive overexpression of star-form microRNAs. PLoS ONE.

[29] Nicolas FE, Hall AE, Csorba T, Turnbull C, Dalmay T (2012). Biogenesis of Y RNA-derived small RNAs is independent of the microRNA pathway. FEBS Lett..

[30] Beitzinger M, Meister G (2011). Experimental identification of microRNA targets by immunoprecipitation of Argonaute protein complexes. Methods Mol. Biol..

[31] Steck E (2007). Chondrocyte secreted CRTAC1: a glycosylated extracellular matrix molecule of human articular cartilage. Matrix Biol..

[32] Tsang J, Zhu J, van Oudenaarden A (2007). MicroRNA-mediated feedback and feedforward loops are recurrent network motifs in mammals. Mol. Cell.

