# Supplementary material for: The role of microRNA-3085 in chondrocyte function
Source: Sci Rep. 2020 Dec 14;10:21923. doi: 10.1038/s41598-020-78606-6 (PMC7736321; doi:10.1038/s41598-020-78606-6)

The role of microRNA-3085 in chondrocyte function
(supplementary information)

Linh Le[1,3,4], Lingzi Niu[1,4], Matthew J Barter[2], David A Young[2], Tamas Dalmay[1], Ian M Clark[1,5]*, Tracey E Swingler[1,5]

[1]Biomedical Research Centre, School of Biological Sciences, University of East Anglia, Norfolk, UK.

[2]Institute of Genetic Medicine, Newcastle University, Newcastle-upon-Tyne, UK.

[3]Current address: Biotechnology Department, Ho Chi Minh City Open University, Vietnam

[4]These two authors contributed equally to the research
[5]These two authors supervised the research equally

Supplementary Information

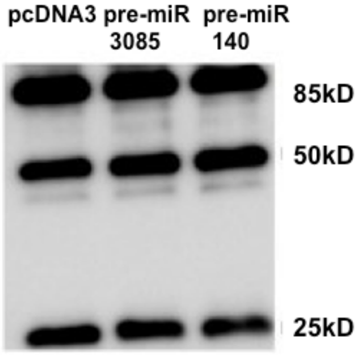

Supplementary Figure 1: SW1353 cells were transiently transfected with either pre-miR-3085 or pre-miR-140 for 48 hours, cell lysates were immunoprecipitated using an Ago2 antibody (see Methods). Western blot using anti-Ago2 (85kD) demonstrates even loading with IgG at 50kD heavy chain and 25kD light chain.

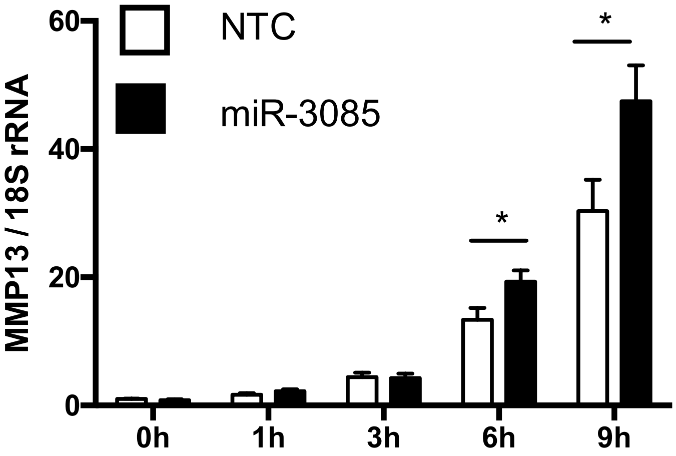

Supplementary Figure 2: Primary human articular chondrocytes were grown in monolayer culture and transiently transfected with miR-3085-3p or a non-targeting control (NTC) for 24 hours prior to stimulation with IL-1β (5ng/ml) or control across a 9 hour time course. MMP13 was measured by qRT-PCR. Mean +/- SEM, n=3; Student's t-test; *, $p<0.05$.

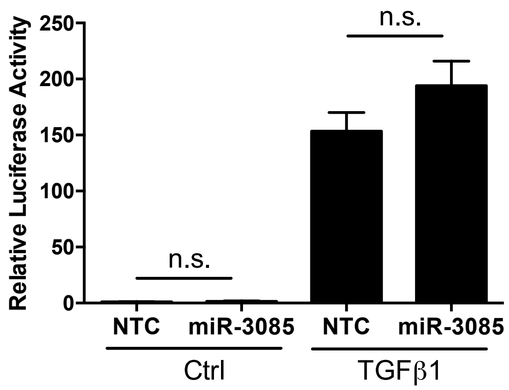

Supplementary Figure 3: SW1353 cells were transfected with an $(CAGA)_{12}$ luciferase reporter +/- miR-3085-3p mimic or non-targeting control (NTC) for 24 hours prior to stimulation with TGFβ1 (4ng/ml) or control for 8 hours. Cell lysates were assayed for luciferase as Methods. Mean +/- SEM, n=3; Student's t-test.

Supplementary Table 1: Primers and probe sequences

qRT-PCR primers and probes			
Gene	Primer	Sequence 5'- 3'	Probe
18s rRNA	F	GCCGCTAGAGGTGAAATTCTTG	5'-FAM-ACCGGCGCAAGACGGA-TAMRA-3'
	R	CATTCTTGGCAAATGCTTTCG	
MMP13	F	AAATTATGGAGGAGATGCCCATT	5'-FAM-CTACAACTTGTTTCTTGTTGCTGCGCATGA-TAMRA-3'
	R	TCCTTGGAGTGGTCAAGACCTAA	
ACAN	F	AAGCACTGGAGTTCTGTGAATCT	#1 (Roche Universal ProbeLibrary)
	R	CGGCATAGCACTTGTCCAG	
COL2A1	F	CCCTGGTCTTGGTGGAAAC	#65 (Roche Universal ProbeLibrary)
	R	TCCTTGCATTACTCCCAACTG	
SOX9	F	GTACCCGCACTTGCACAAC	#61 (Roche Universal ProbeLibrary)
	R	TCTCGCTCTCGTTCAGAAGTC	
ID1	F	CCAGAACCGCAAGGTGAG	#39 (Roche Universal ProbeLibrary)
	R	GGTCCCTGATGTAGTCGATGA	
MYD88	F	GGACCCAGCATTGAGGAG	#18 (Roche Universal ProbeLibrary)
	R	ACAGCGGCCACCTGTAAA	
AXIN2	F	GCTGACGGATGATTCCATGT	#56 (Roche Universal ProbeLibrary)
	R	ACTGCCCACACGATAAGGAG	

3'UTR construct cloning primers		
Gene	Primer	Sequence 5'- 3'
ACAN	F	GCTCGCTAGCCTCGA CGTTTGTATTCCCTTCCTGC
	R	CGACTCTAGACTCGA CTCTGATGGCTCTCTCCTGC
COL2A1	F	GCTCGCTAGCCTCGA GGGAGAAGACGCAGAGCG
	R	CGACTCTAGACTCGA TGGACCTTACCAAAGGAGG
SMAD3	F	GCTCGCTAGCCTCGA GAGGACACAGGAAGAGACGG
	R	CGACTCTAGACTCGA GTGTTTCTGGATGCTGAGCC
SMAD4	F	GCTCGCTAGCCTCGATGCTCCCTTCGTTATTGCC
	R	CGACTCTAGACTCGA GCCACAGACTTTAACCAGGC
MYD88	F	GCTCGCTAGCCTCGAAGACTGTTCTGAGGCCCTGG
	R	CGACTCTAGACTCGAGGAAGAATGGCAAATATCGGCT

3'UTR QuikChange primers		
Gene	Primer	Sequence 5'- 3'
ACAN site1	F	GGTAGACCGTTCTCACGCACTAGTTGTCCCCACCGCAGGCAGGC
	R	GCCTGCCTGCGGTGGGGACAACTAGTGCGTGAGAACGGTCTACC
ACAN site2	F	CCTGCGTTGTGCTGGTTTCAGAGGAATTCCTGCCCCAGAGGGACTGACATTT
COL2A1	R	ACCGCGGGGCACTAGTGAGCCGGGCCCGGGCGGA
COL2A1	F	TCCGCCCGGGCCCGGCTCACTAGTGCCCCGCGGT

Figure 3E

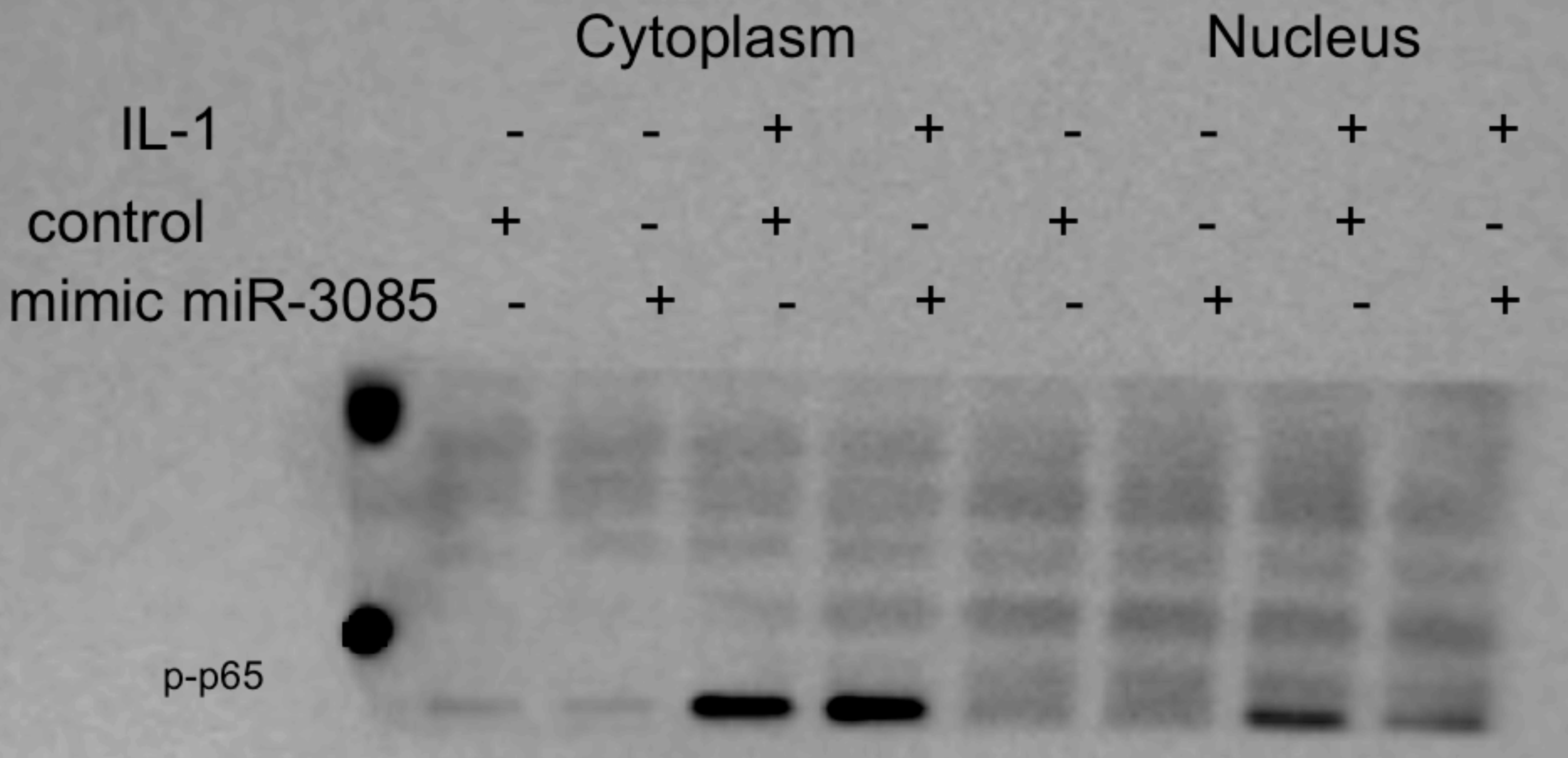

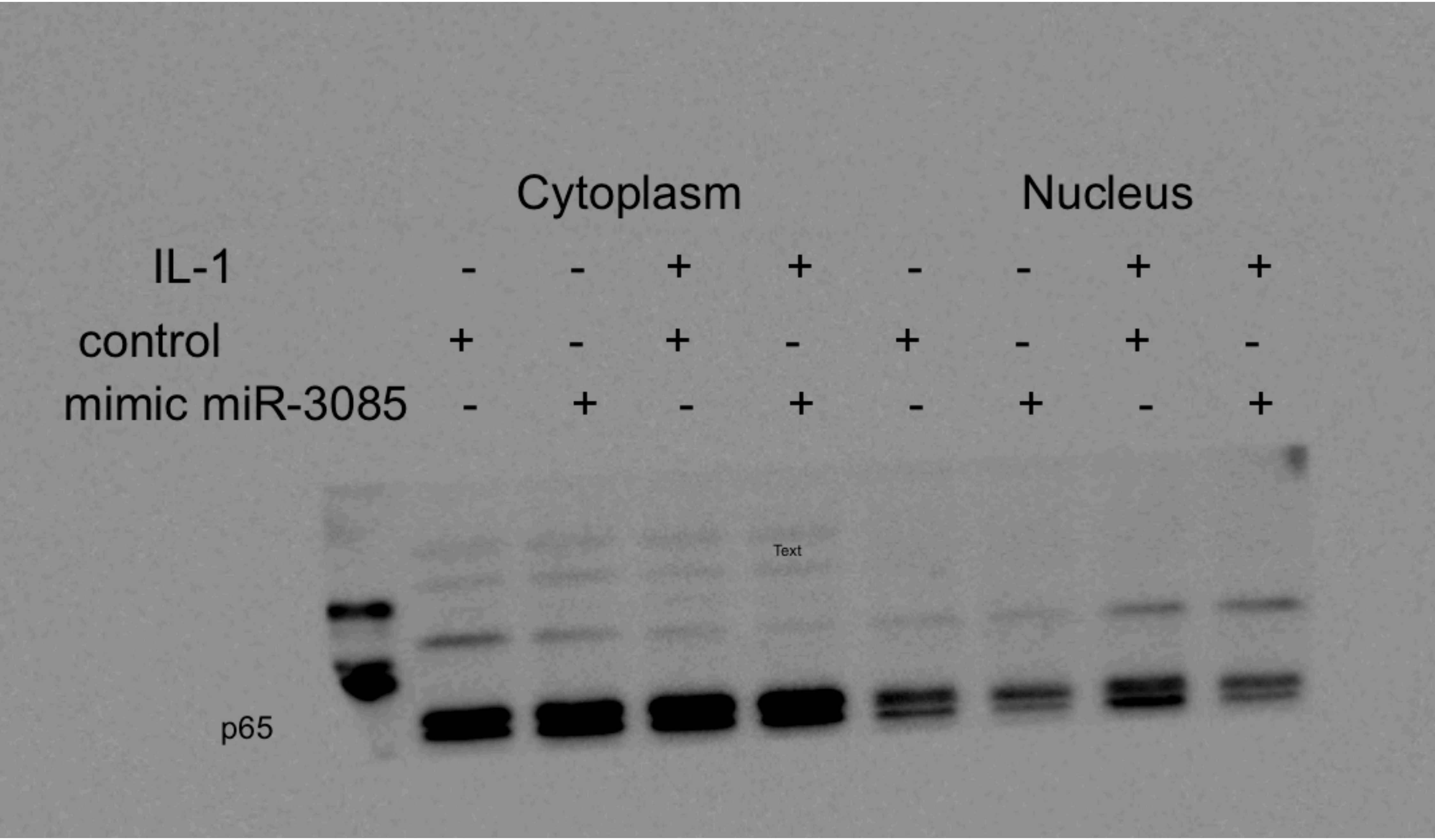
Cytoplasm
Nucleus
IL-1 - - + + - - + +
control + - + - + - + -
mimic miR-3085 - + - + - + - +
Text
p65

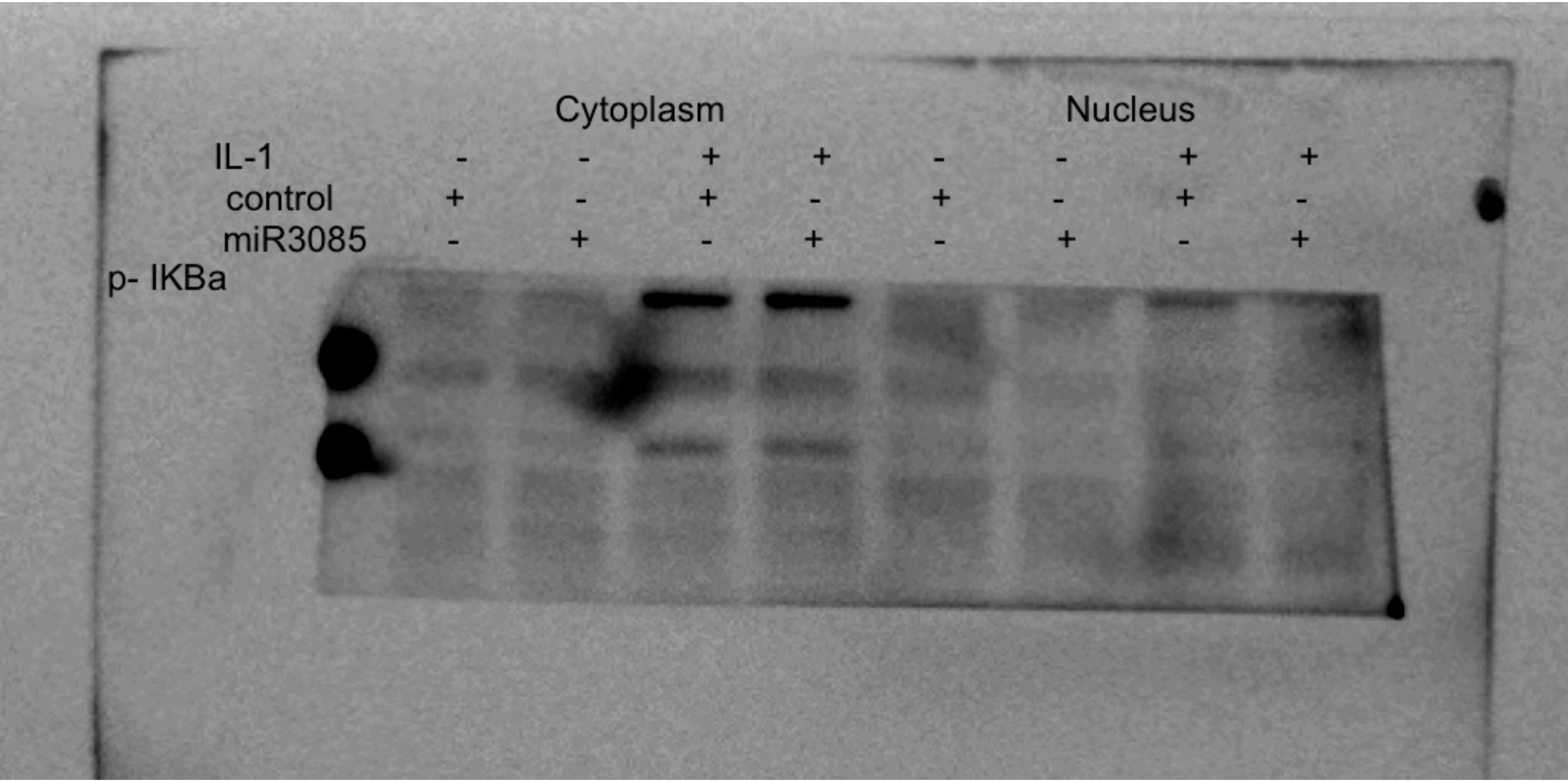

Cytoplasm
Nucleus
IL-1 - - + + - - + +
control + - + - + - + -
miR3085 - + - + - + - +
p- IKBa

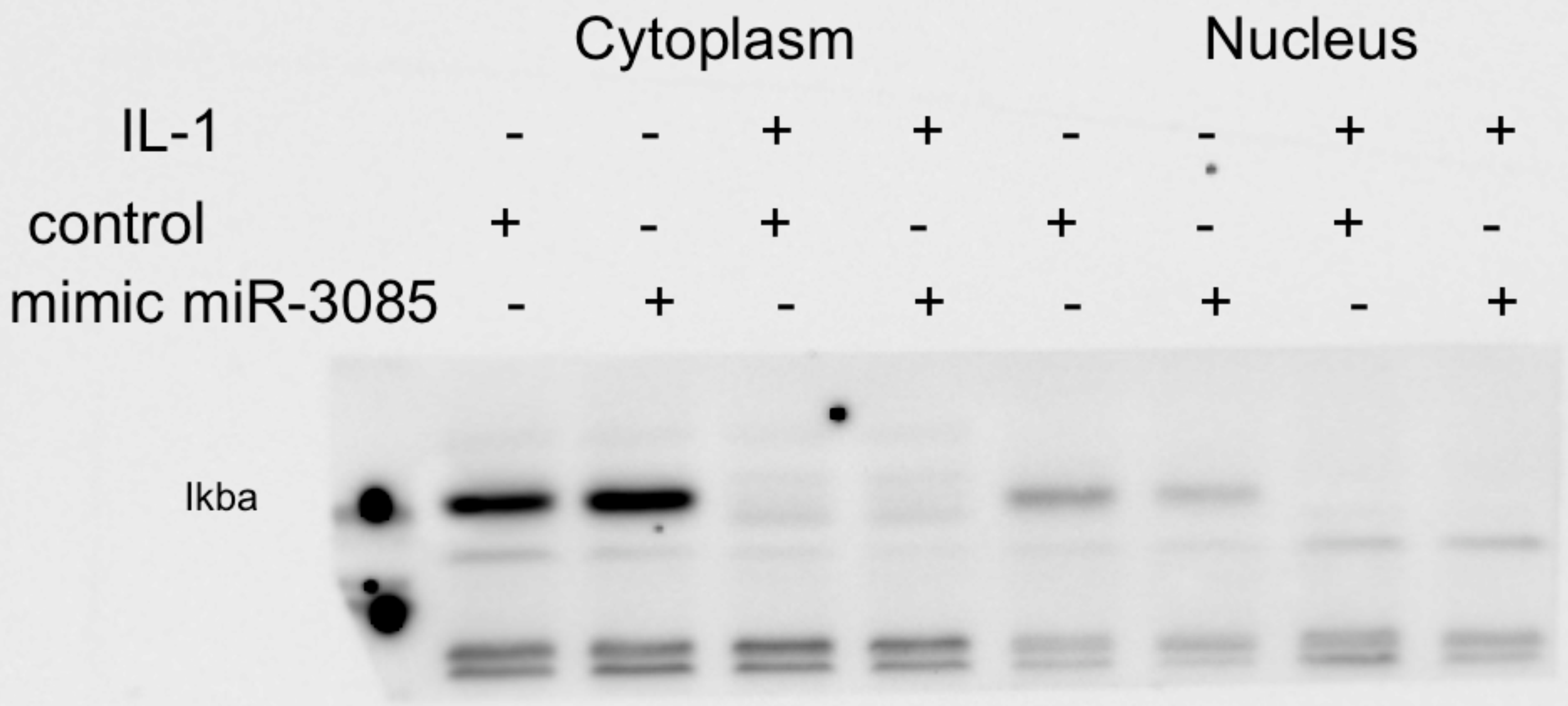
Cytoplasm
Nucleus
IL-1 - - + + - - + +
control + - + - + - + -
mimic miR-3085 - + - + - + - +
Ikba

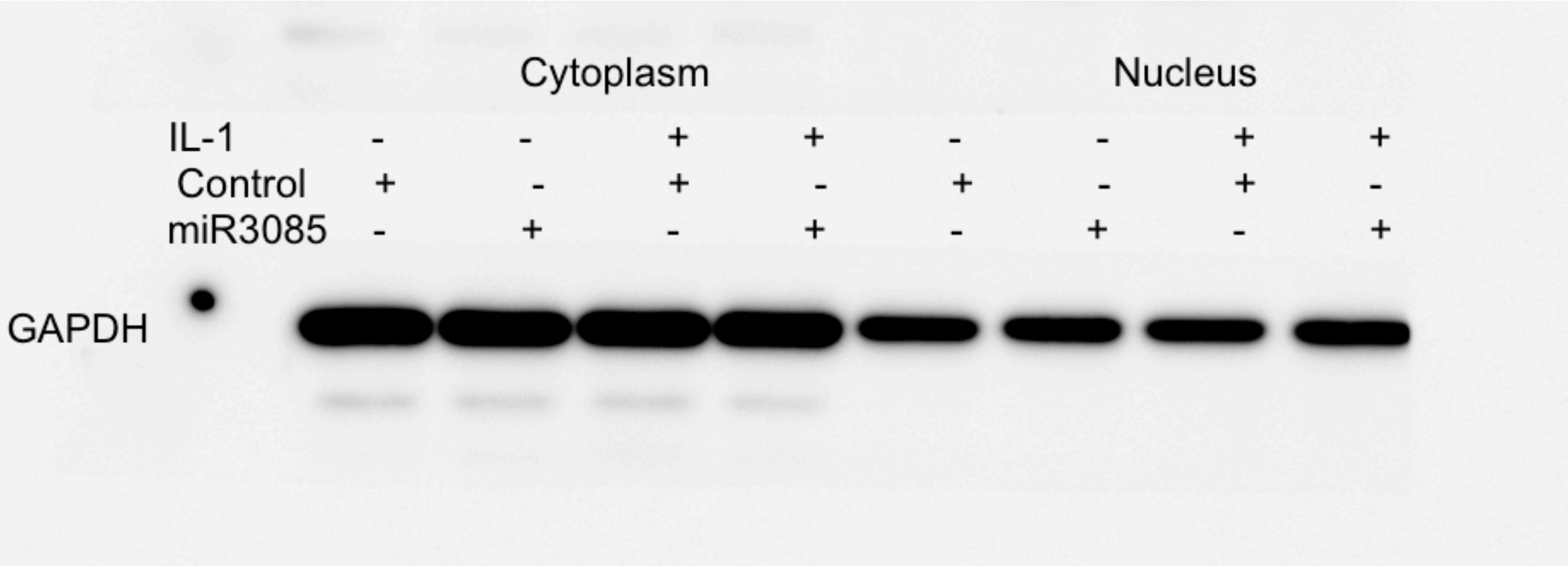
Cytoplasm
Nucleus
IL-1 - - + + - - + +
Control + - + - + - + -
miR3085 - + - + - + - +
GAPDH

Figure 4C

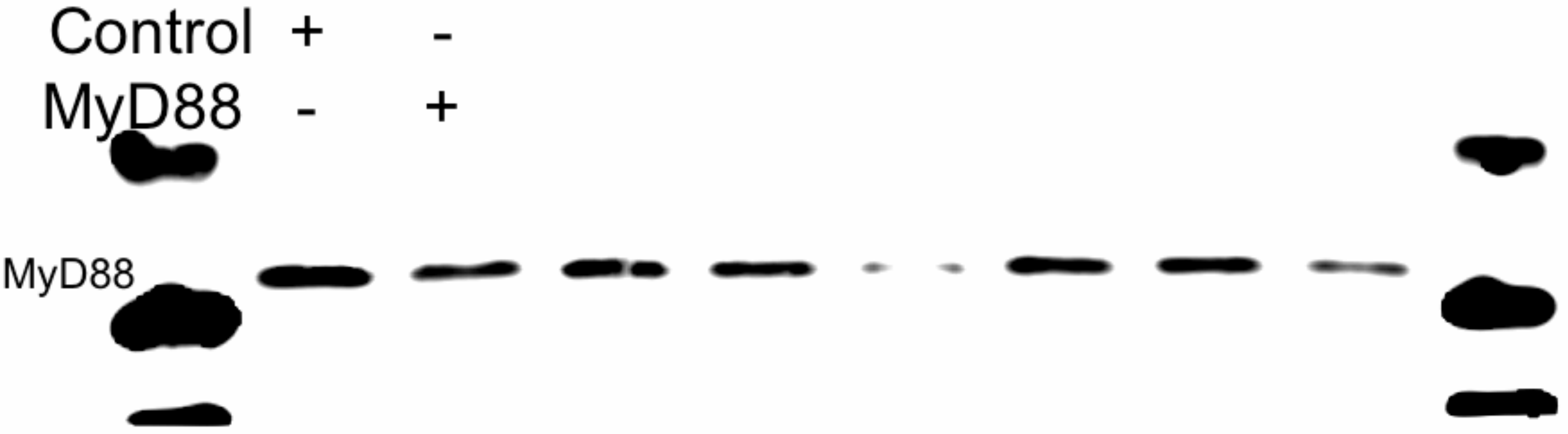

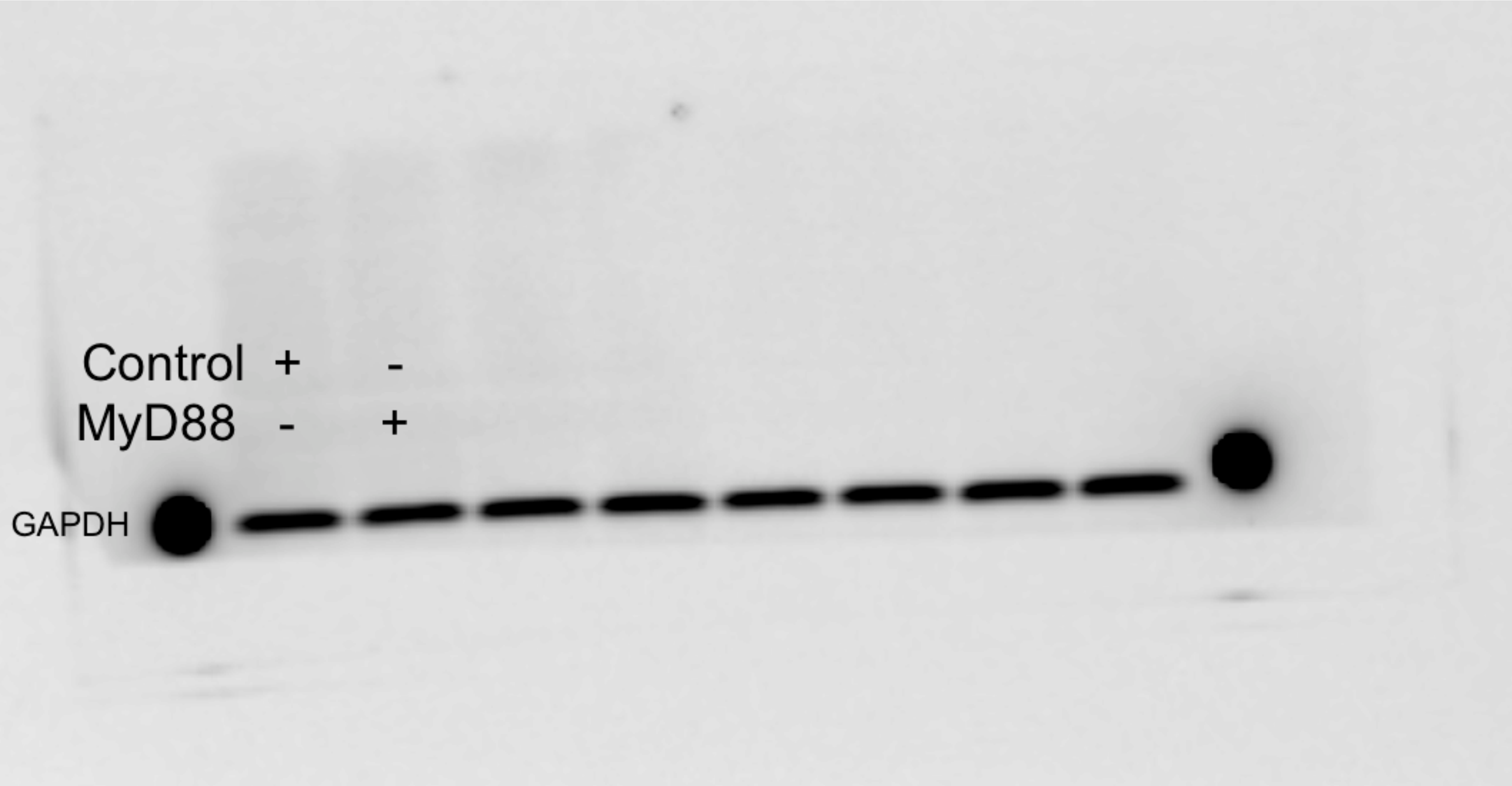
Control + -
MyD88 - +
GAPDH

Fig 4E

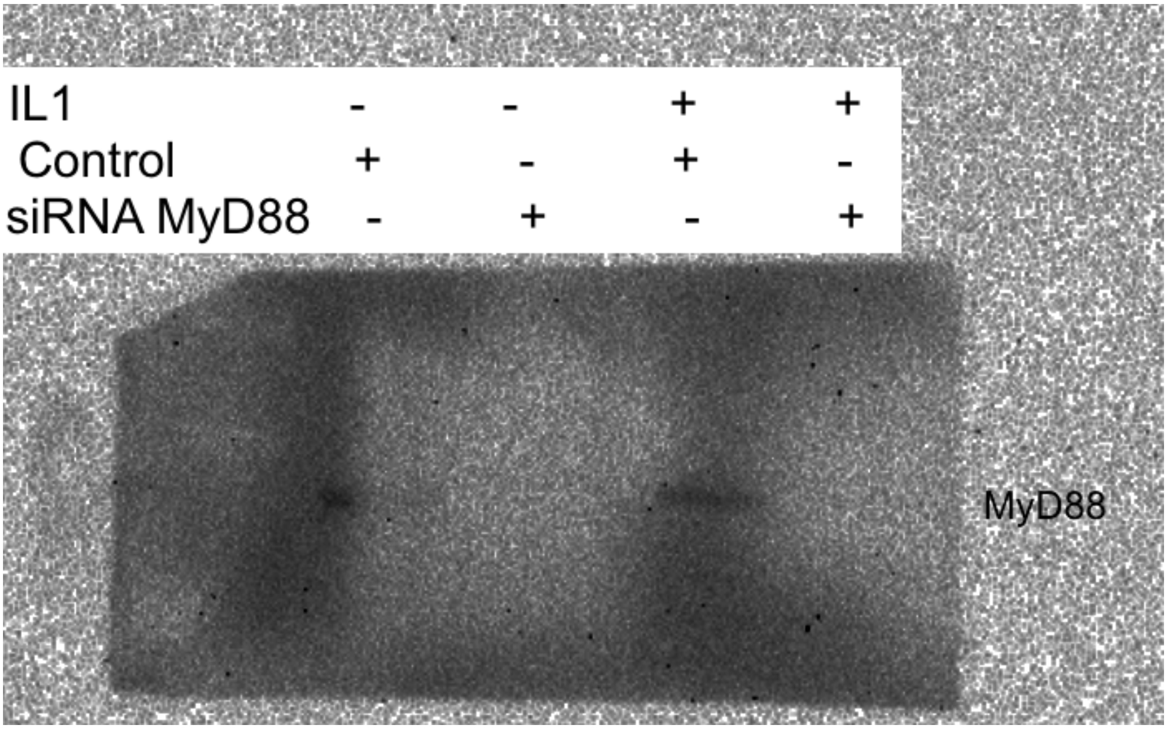

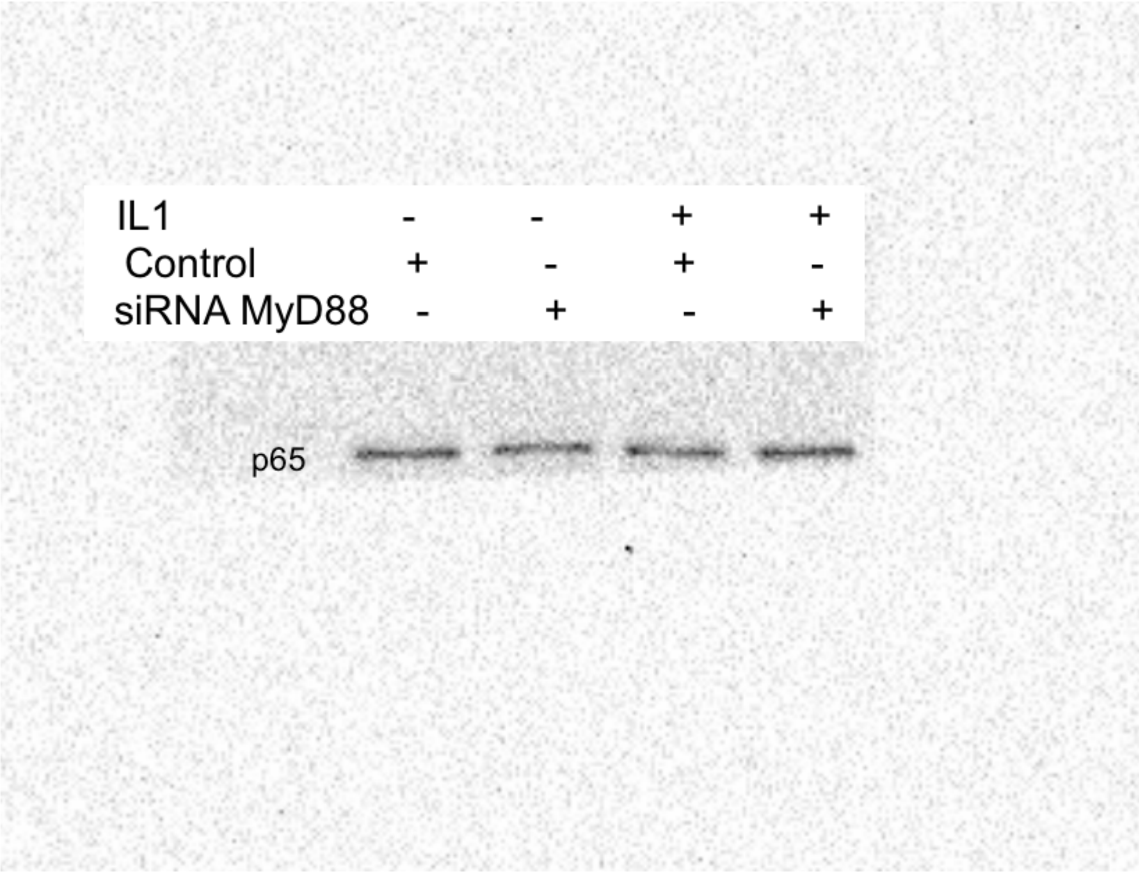
IL1 - - + +
Control + - + -
siRNA MyD88 - + - +
p65

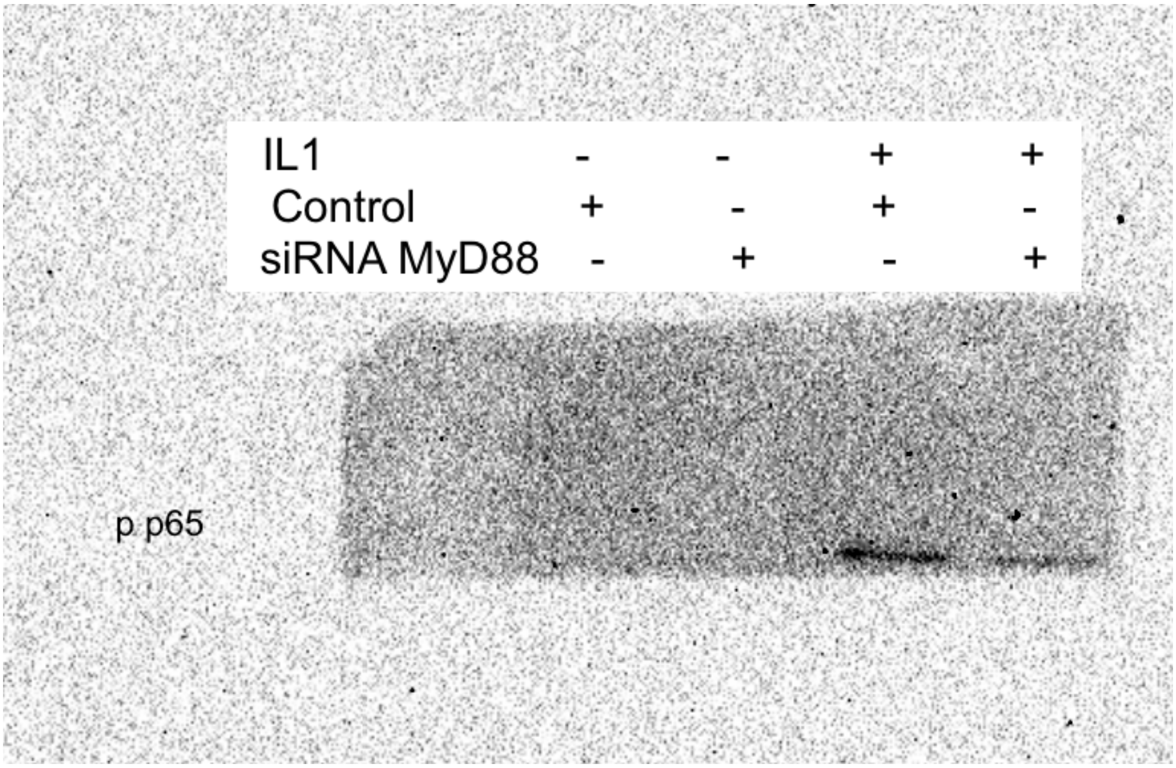
IL1 - - + +
Control + - + -
siRNA MyD88 - + - +
p p65

Figure 5F

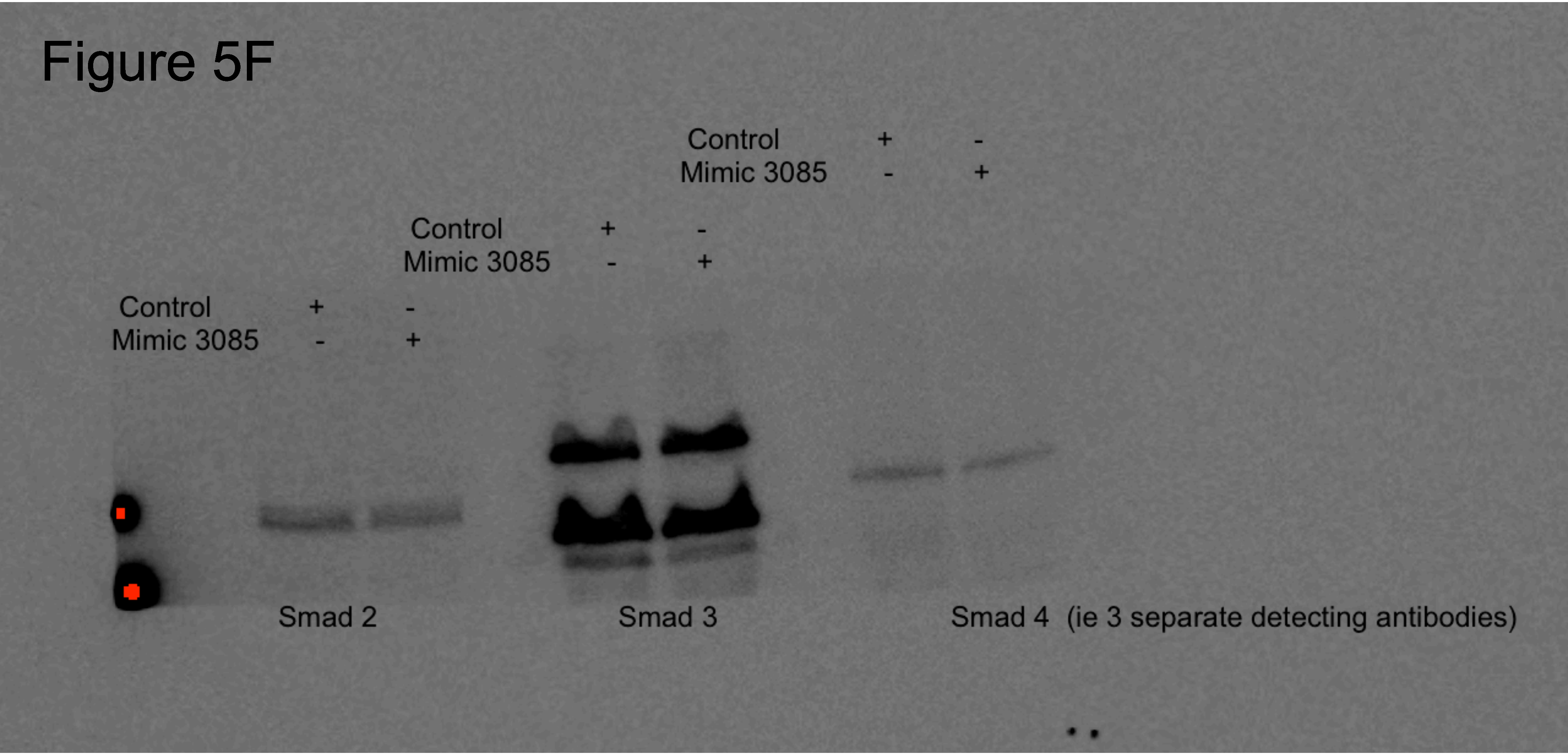

Control + -
Mimic 3085 - +
Control + -
Mimic 3085 - +
Control + -
Mimic 3085 - +
Smad 2
Smad 3
Smad 4 (ie 3 separate detecting antibodies)

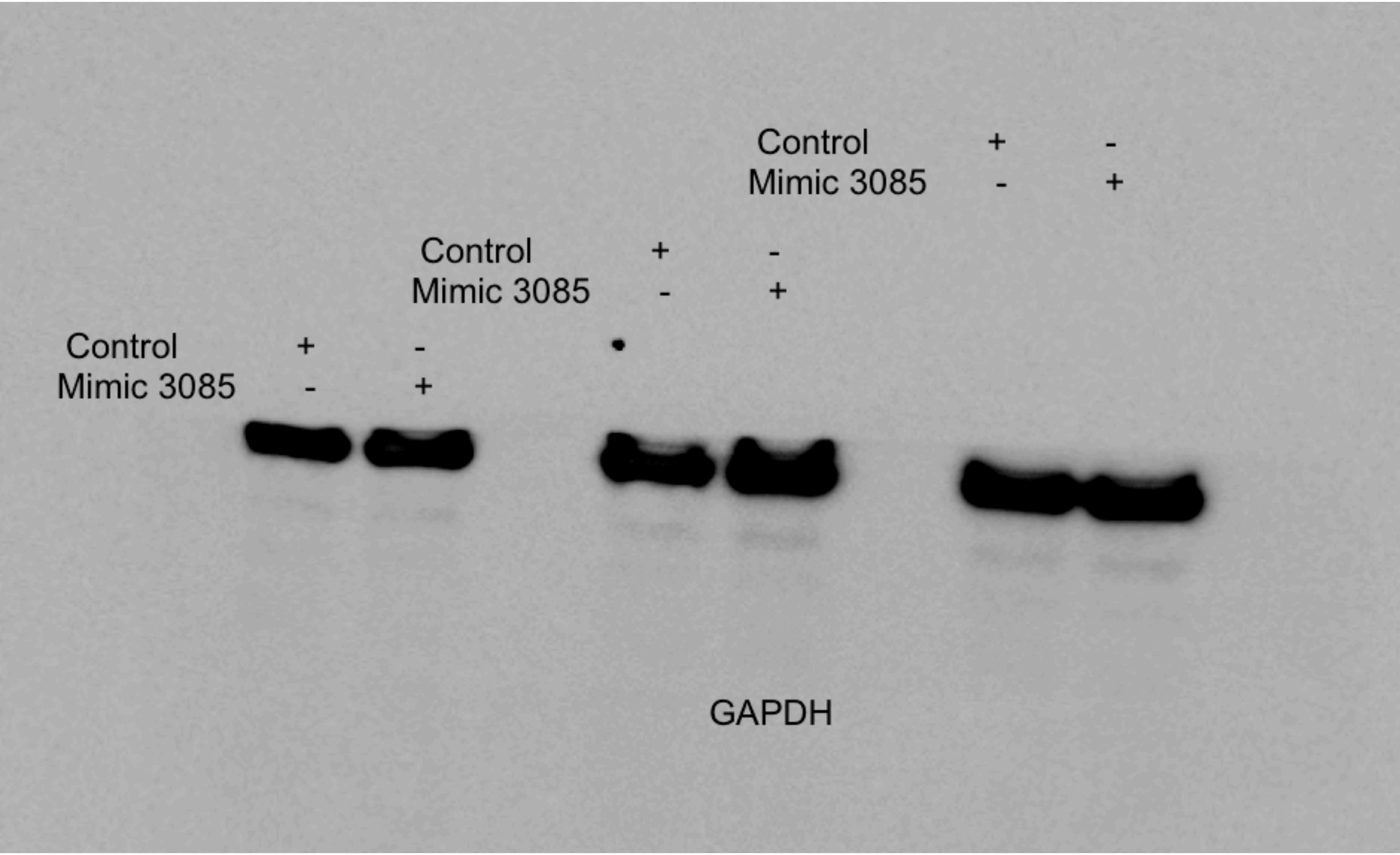
Control + -
Mimic 3085 - +
Control + -
Mimic 3085 - +
Control + -
Mimic 3085 - +
GAPDH

Figure 5G

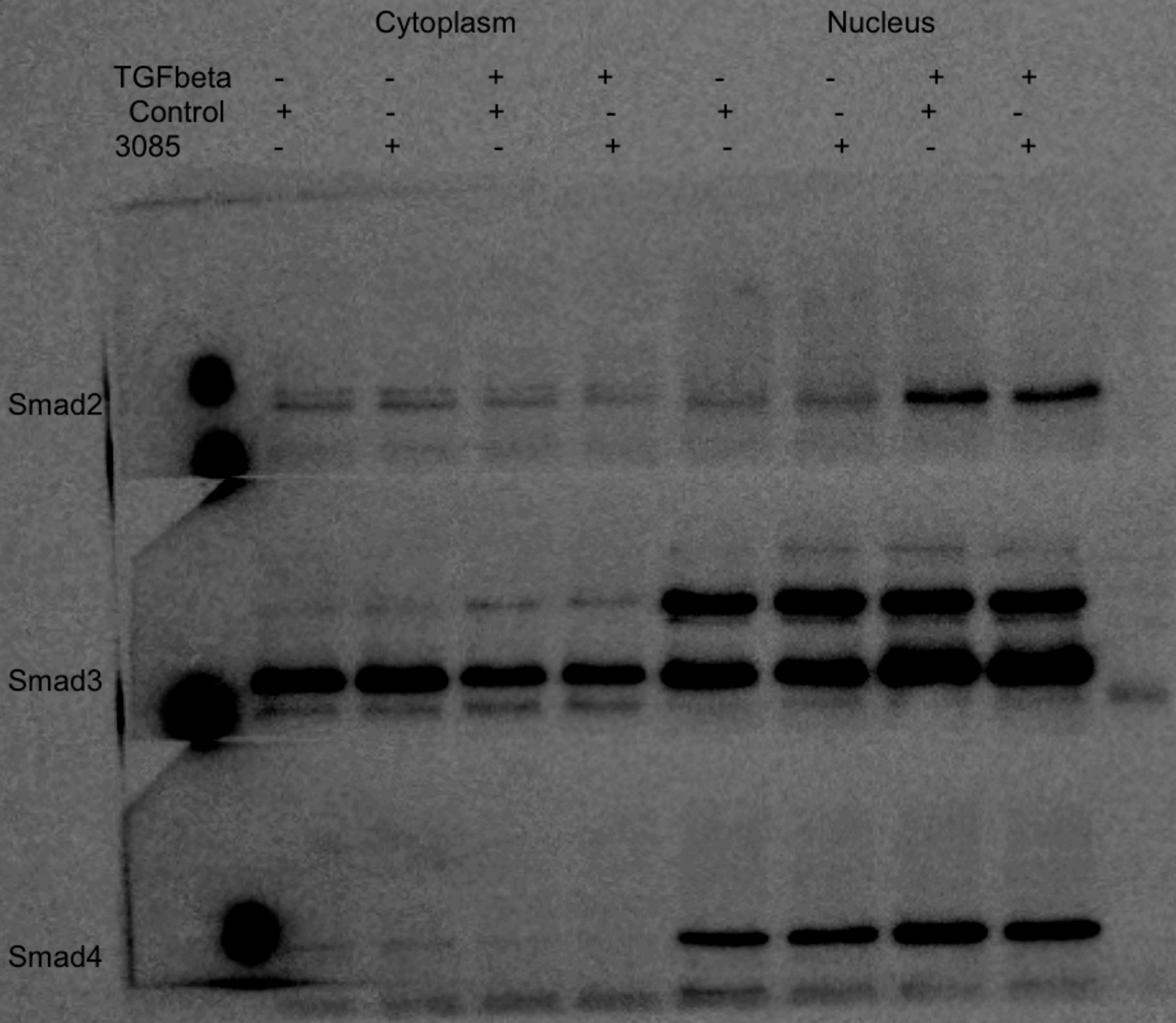

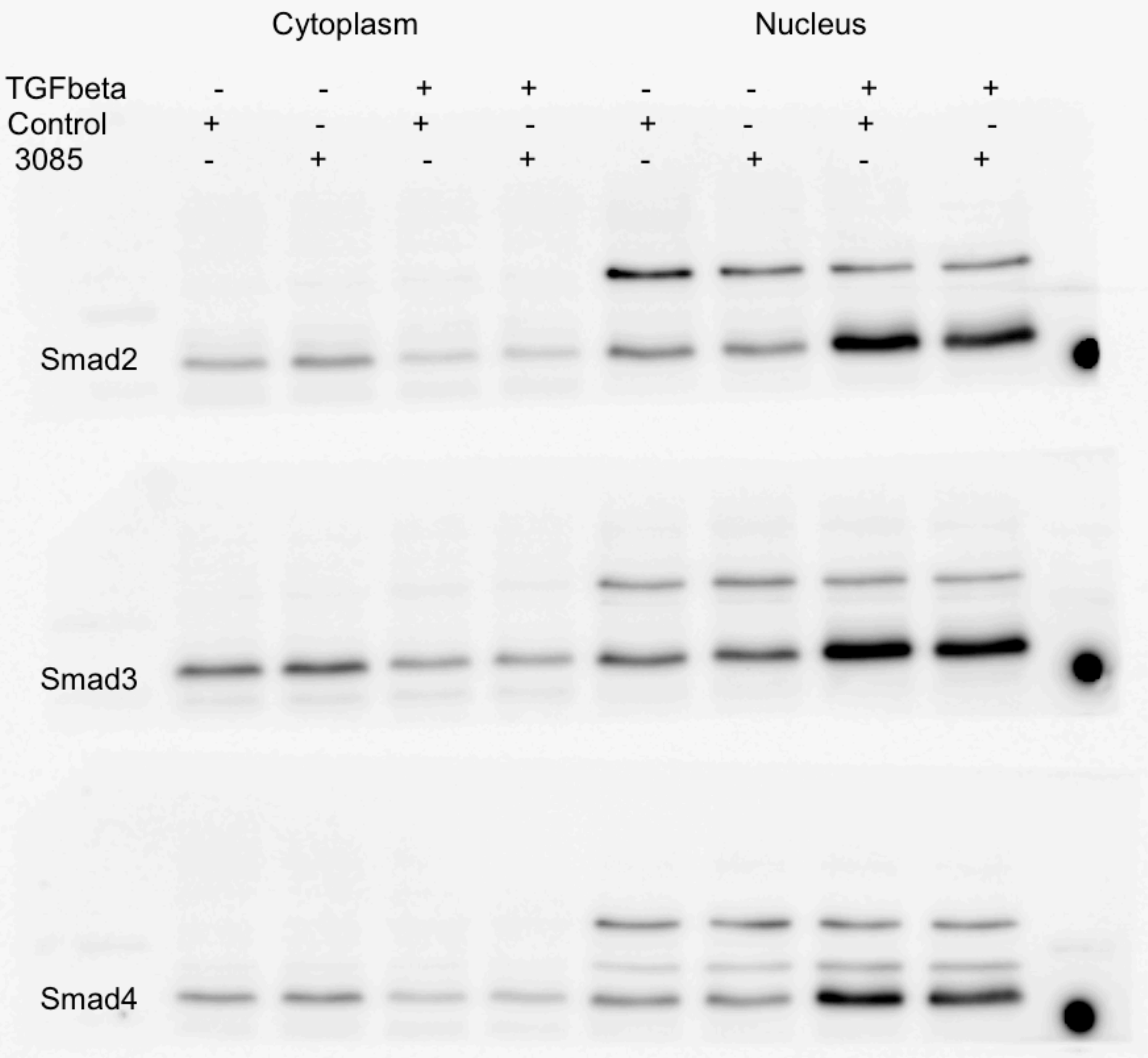
Cytoplasm
Nucleus
TGFbeta - - + + - - + +
Control + - + - + - + -
3085 - + - + - + - +
Smad2
Smad3
Smad4

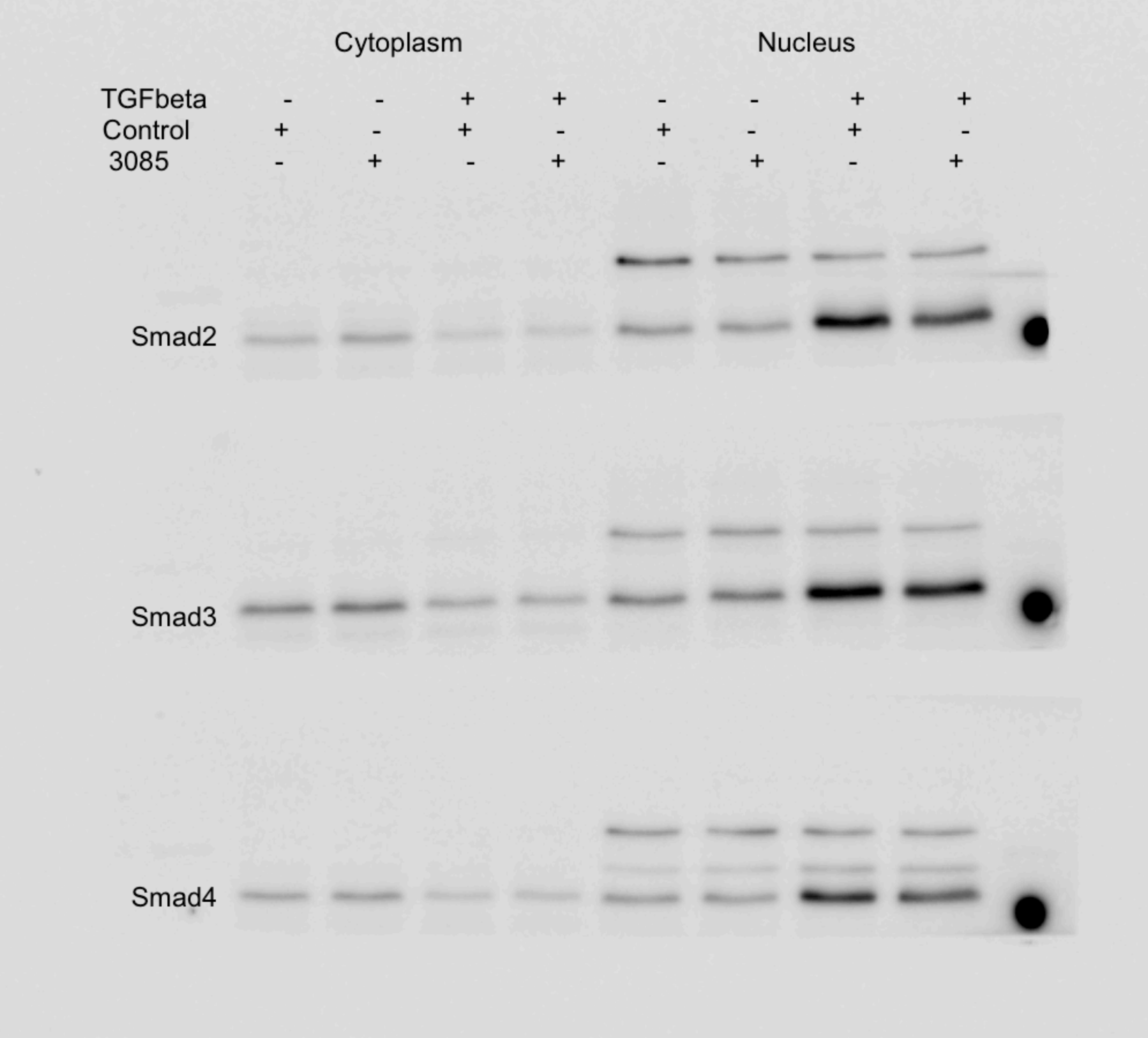
Cytoplasm
Nucleus
TGFbeta - - + + - - + +
Control + - + - + - + -
3085 - + - + - + - +
Smad2
Smad3
Smad4

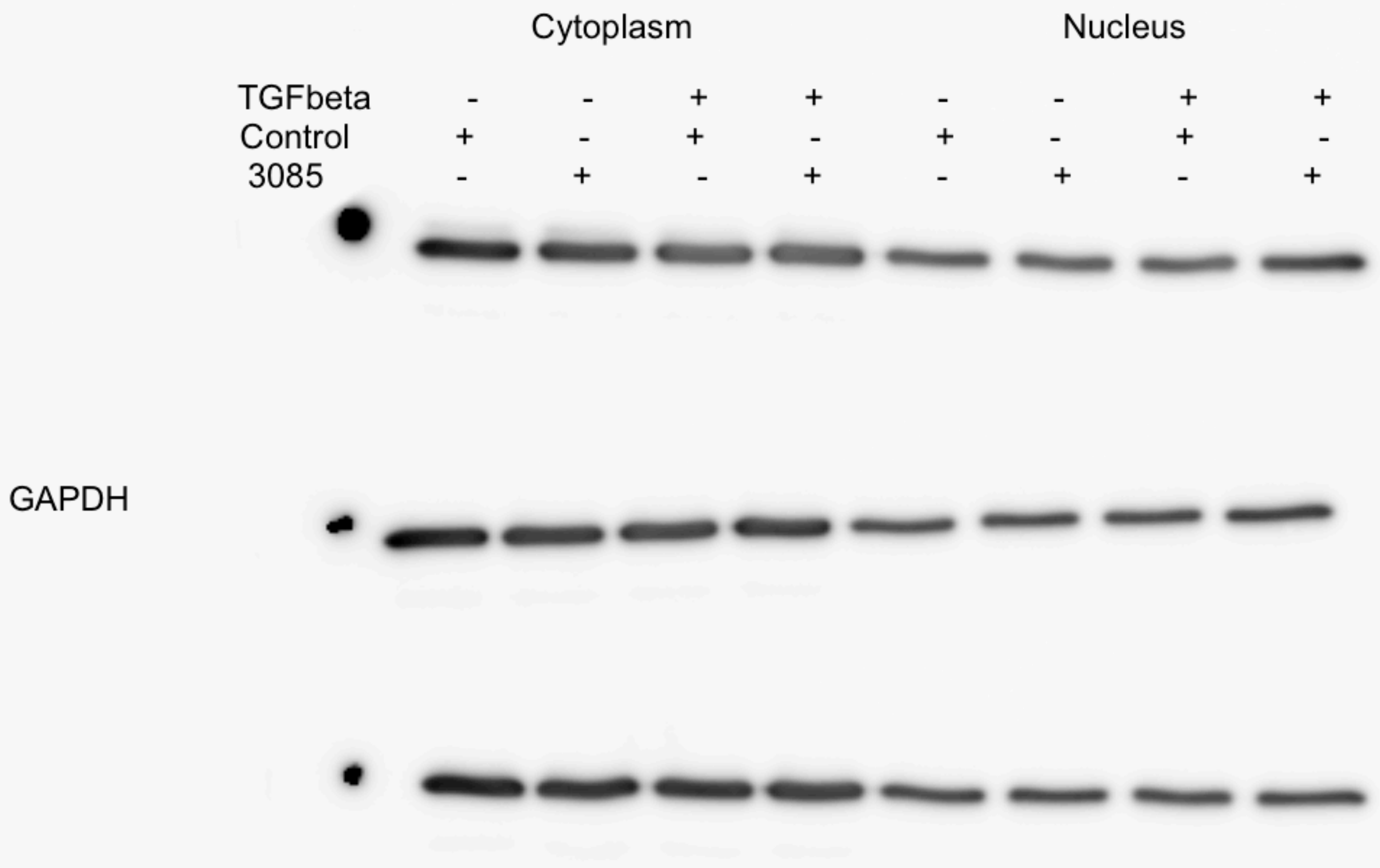
Cytoplasm
Nucleus
TGFbeta - - + + - - + +
Control + - + - + - + -
3085 - + - + - + - +
GAPDH

Figure 5H

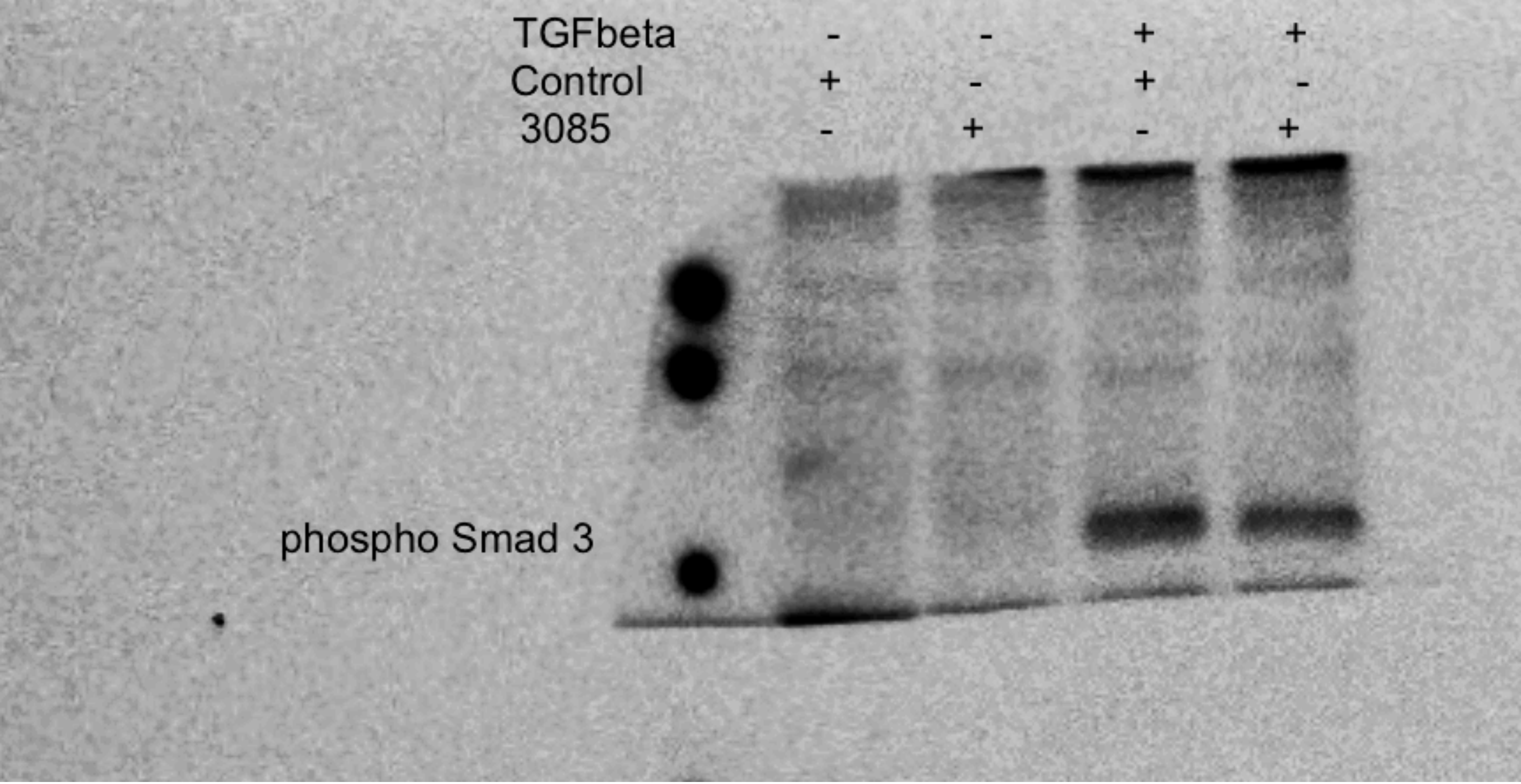

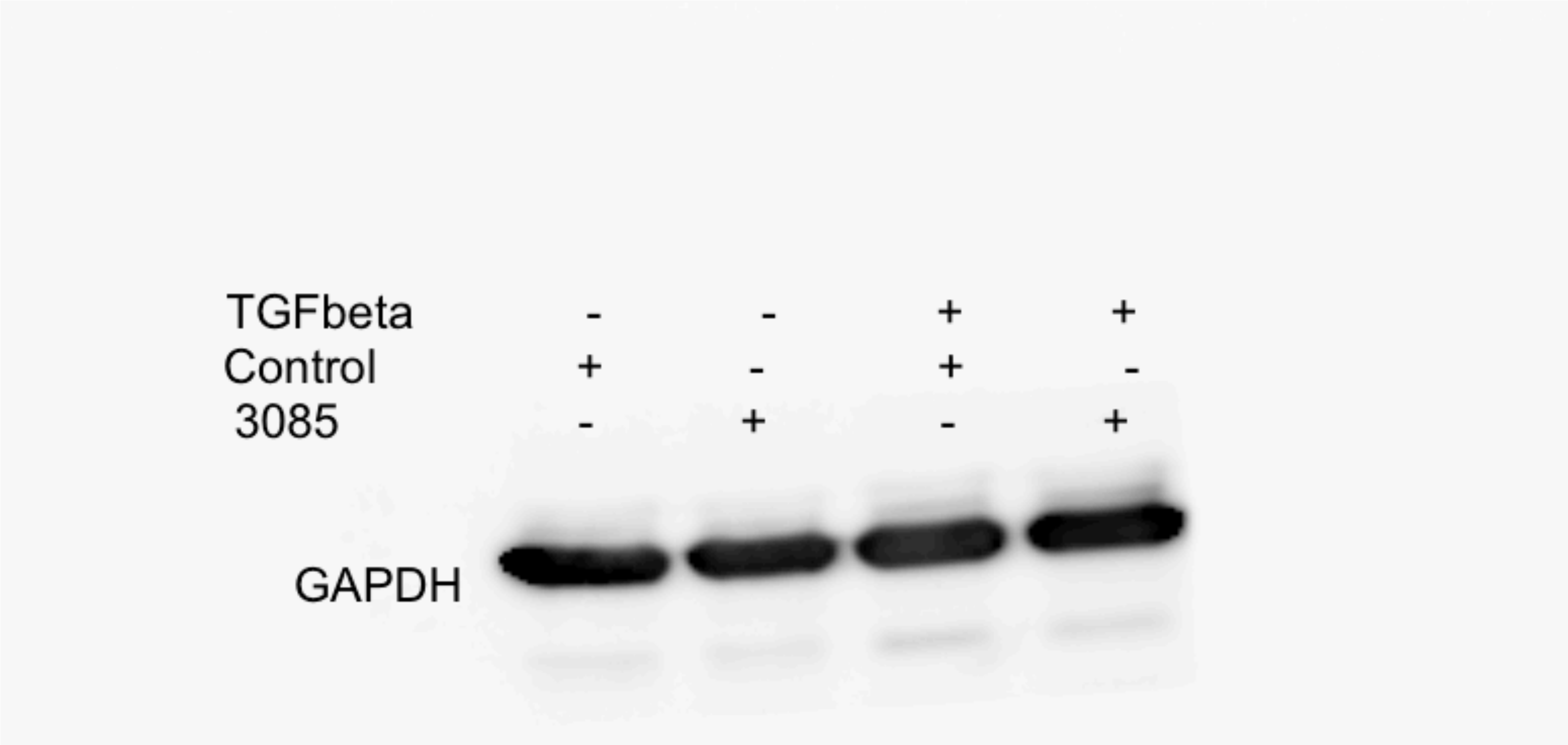
TGFbeta - - + +
Control + - + -
3085 - + - +
GAPDH

Suppl Fig 1

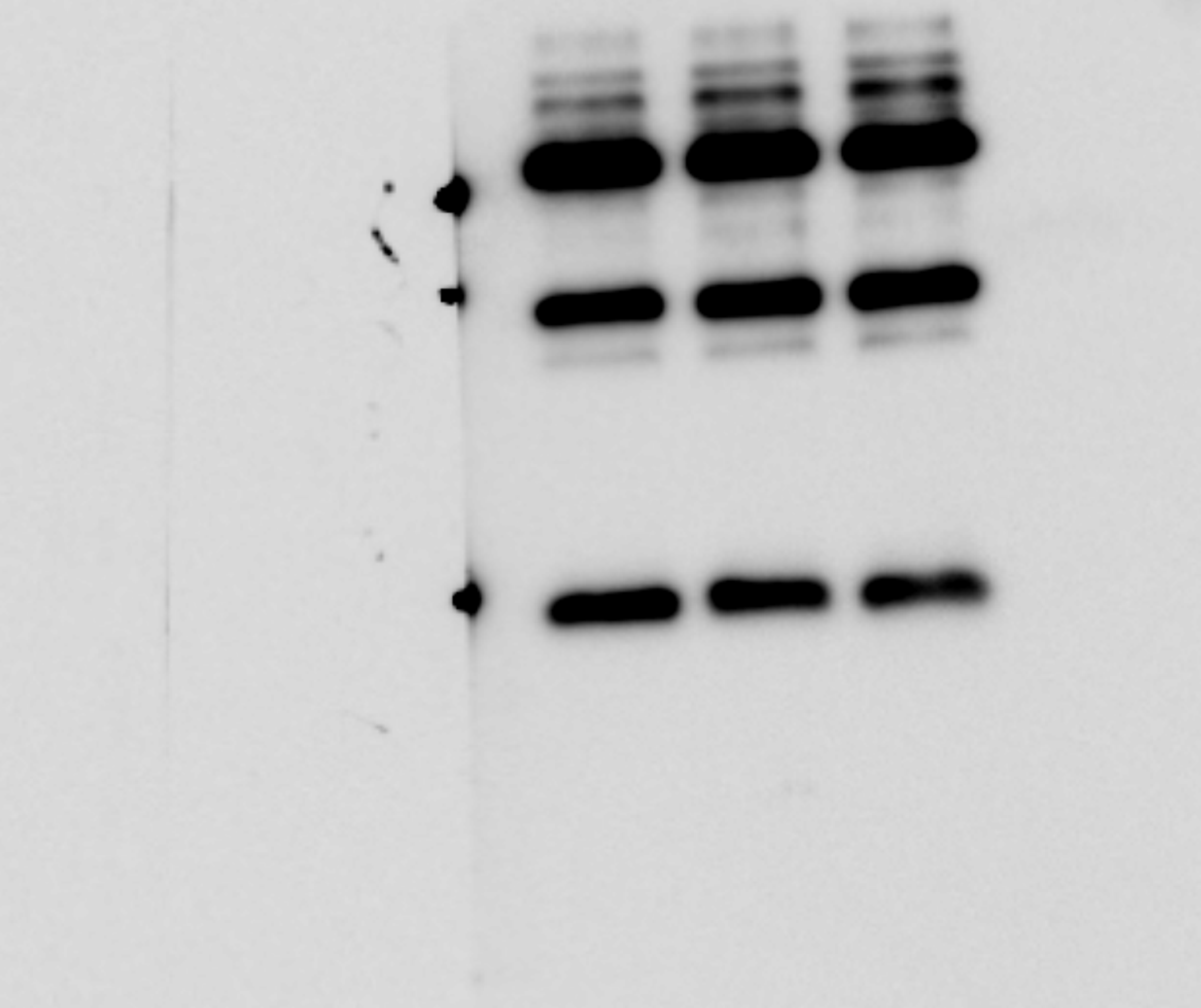

Supplement: Supplementary file 1 — Supplementary information. [file 41598_2020_78606_MOESM1_ESM.pdf]
